# The Many Faces of Engagement: A Scoping Review of Paediatric Patient and Family Engagement in Clinical Care, Education and Research

**DOI:** 10.1111/hex.70756

**Published:** 2026-08-18

**Authors:** Brooke Allemang, Ida Dehmardan, Pranshu Maini, Francine Buchanan, Ivona Novak, Vanessa Carbone, Dalya Kablawi, Lin Li, Linda Nguyen, Kimberly Courtney, Jessie Cunningham, Iveta Lewis, Carla P. Southward, Kristin Cleverley, Sarah Munce, Alene Toulany

**Affiliations:** ^1^ The Hospital for Sick Children Toronto Ontario Canada; ^2^ Child Health Evaluative Sciences SickKids Research Institute Toronto Ontario Canada; ^3^ Factor‐Inwentash Faculty of Social Work University of Toronto Toronto Ontario Canada; ^4^ Faculty of Health Sciences McMaster University Hamilton Ontario Canada; ^5^ Institute of Health Policy, Management and Evaluation University of Toronto Toronto Ontario Canada; ^6^ The Office of Engagement The Hospital for Sick Children Toronto Ontario Canada; ^7^ Neurosciences and Mental Health SickKids Research Institute Toronto Ontario Canada; ^8^ Centre for Addiction and Mental Health Toronto Ontario Canada; ^9^ Lawrence Bloomberg Faculty of Nursing University of Toronto Toronto Ontario Canada; ^10^ Faculty of Social Work University of Calgary Calgary Alberta Canada; ^11^ The CHEO Research Institute Ottawa Ontario Canada; ^12^ The Ontario Child Health Support Unit Ottawa Ontario Canada; ^13^ Holland Bloorview Kids Rehabilitation Hospital Toronto Ontario Canada; ^14^ Temerty Faculty of Medicine University of Toronto Toronto Ontario Canada; ^15^ Bloorview Research Institute Toronto Ontario Canada; ^16^ Rehabilitation Sciences Institute University of Toronto Toronto Ontario Canada; ^17^ Institute for Clinical Evaluative Sciences Toronto Ontario Canada

**Keywords:** advisory committee, patient and family participation, patient and public involvement, patient engagement, patient‐oriented research, paediatrics, scoping review

## Abstract

**Background:**

Engagement of paediatric patients, families and caregivers in clinical care, health research and health education is increasingly recognised as essential to improving the quality, relevance and equity of health services. Despite growing interest in its application across clinical care, research and education domains, engagement practices remain poorly connected, inconsistently defined and unevenly implemented. A system‐wide understanding of engagement is needed to clarify common principles, highlight gaps in practice and ensure that approaches are aligned across domains—conditions that are necessary for coherence and equity.

**Objective:**

To identify the commonalities and distinctions between paediatric patients and family engagement in clinical care, education and research contexts in paediatric healthcare institutions.

**Methods:**

Following PRISMA‐ScR guidelines and the Joanna Briggs Institute framework, a scoping review was conducted across five databases and eleven grey literature sources. Eligible articles involved paediatric patients (0–24 years old), families or caregivers involved in engagement activities related to clinical care, research or education. Three youth and family partners contributed to all review stages, including protocol refinement, screening, extraction, interpretation and manuscript preparation.

**Results:**

Of 16,817 records screened, 113 studies met inclusion criteria. Commonalities emerged across domains, including the predominance of small engagement groups, the frequent use of co‐development and feedback roles for partners, and a shared tendency to operate at the *Collaborate* level of the International Association for Public Participation Spectrum. The application of equity, diversity and inclusion principles in engagement and the impacts of engagement were rarely described across domains. Notable distinctions identified included differences in terminology, evaluation practices and reporting of engagement.

**Discussion and Conclusion:**

This review identified commonalities and distinctions in how paediatric engagement is conceptualised and enacted across clinical care, research and education, including in partner responsibilities, engagement methods and outcomes. Inconsistent reporting, variable evaluation practices and imprecise terminology reduce comparability across contexts and limit the transferability of engagement practices across the domains of paediatric clinical care, research and education.

**Patient or Public Contribution:**

This review was co‐designed and performed in collaboration with three youth and family partners who actively participated across all phases of the research process from conceptualisation, study selection and screening to data analysis and interpretation, and manuscript preparation.

## 1 Introduction

Patient engagement is the meaningful and active collaboration between patients, families, clinicians, researchers and decision‐makers to ensure health systems reflect the needs of those who use them, supporting high‐quality and equitable care [1]. In paediatrics, patient engagement is essential for delivering patient‐centred healthcare and for designing systems that are responsive to the needs of children, youth and families; however, it also requires developmentally appropriate approaches that balance a young person's emerging autonomy with meaningful caregiver involvement [2]. Paediatric engagement approaches must also consider the triadic relationship between the child, caregiver, and clinician or researcher, as well as address the legal and ethical constraints that often limit children's autonomy [2, 3, 4, 5, 6]. While a substantial body of evidence describes methods and frameworks for engaging patients and families in healthcare, the literature remains siloed across clinical care, research and education and is predominantly oriented towards adult patient engagement. The existing evidence does not adequately capture the unique needs and perspectives of children, youth, families and caregivers; it offers limited insight into engagement methods that are developmentally appropriate for young people, how their interest in engaging may change over time, and the specific areas of the healthcare system in which they wish to be involved.

In clinical settings, paediatric patient and family engagement often focuses on family‐centred care, shared decision‐making and promoting child autonomy [7, 8]. Family‐centred care models have long emphasised the importance of treating families as partners in paediatric care [2]. Youth and families may inform models of clinical care, help develop decision aids to weigh treatment options, assist in revising communication pathways or co‐design interventions aimed at supporting more patient‐oriented care—practices shown to support better outcomes, higher satisfaction and improved safety [9, 10]. In education, youth and families contribute as co‐educators in health professional training, shape patient‐facing materials and help develop curricula [11]. These contributions can add relevance, authenticity and emotional insight to learning environments [12]. In research, engagement can include youth and family roles on advisory boards, co‐design of study materials or involvement across the research cycle, from setting priorities to sharing results [11, 13, 14]. Research partnerships have been found to result in more meaningful, ethical and applicable studies, while also enhancing recruitment and retention [15, 16].

Despite the widespread use of engagement practices across clinical care, health research and health education, existing reviews tend to examine these domains in isolation or in parallel, rather than exploring their interconnections. This siloed approach leaves critical questions unanswered: Where do engagement methods align? Where do they diverge? And what lessons could be shared across settings to enhance impact? When engagement literature remains fragmented by context, opportunities for integration, infrastructure development and more coordinated strategies are missed. Patients and families often serve as the connecting thread across these domains. They navigate multiple systems of engagement, using their lived experience to inform improvements in care, research and education. Yet, limited research has examined how engagement is operationalised across these three areas within paediatric institutions. To address this gap, a scoping review was conducted to systematically map the published and unpublished (grey) literature on paediatric patient and family engagement in clinical care, education and research. Our guiding question was: What are the commonalities and distinctions between paediatric patient and family engagement approaches when applied in clinical care, education and research contexts?

## 2 Methods

A scoping review of the published and unpublished literature was conducted in collaboration with three youth and family partners, following PRISMA‐ScR guidelines [17], the Joanna Briggs Institute framework [18] and guidance on engaging knowledge users [19]. The study methods are described in detail in the published protocol [20].

### 2.1 Search Strategy

An initial limited search was conducted by the first author to identify seminal and exemplar articles focused on the topic. Words contained in the titles, abstracts and keywords of these articles were used to inform the search strategy. The full search strategy was developed by the team alongside a professional medical librarian (J.C.) and executed in November 2024 in the following databases: Ovid MEDLINE(R) ALL, Ovid Embase+Embase Classic, Ovid EBM Reviews Cochrane Central Register of Controlled Trials, Ebscohost CINAHL Plus and Clarivate Web of Science. Results were limited to English‐language articles published since 2011, the year that Canada's Strategy for Patient‐Oriented Research [1] was established. Non‐primary studies, conference abstracts, protocols and narrative reviews were excluded. Backward citation searching was not conducted in this review due to resource constraints, given the volume of relevant citations identified through database searches. The grey literature search was conducted by a secondary health sciences librarian (I.L.) in September 2025, to complement the traditional biomedical resources in order to reduce the risk of publication bias [21]. The grey literature search strategy was informed by the MEDLINE search strategy, and the final search terms were refined in collaboration with the research team. See Appendix A for the detailed search strategy. The grey literature search included Web of Science, Scopus, CADTH, RAND, Cerebral Palsy Research Network, CIHI, Health Canada, HQO, DuckDuckGo, Oister, OpenDoar, ProQuest and Google Scholar.

### 2.2 Evidence Screening and Selection

Articles were collated and imported into Covidence Systematic Review Software, and duplicates were removed. A two‐level screening of the academic literature was conducted by seven members of the research team (B.A., I.D., D.K., L.L., P.M., L.N. and C.S.). The grey literature was screened by two members of the research team (I.D. and V.C.). Level 1 screening of titles and abstracts against inclusion/exclusion criteria was conducted by at least two reviewers independently. Inclusion criteria were: (1) relevant to paediatric health or mental health institutions; (2) examined patient, family or caregiver (PFC) engagement; (3) focused on health or mental healthcare; and (4) involved a paediatric population (0–24 years old). Our youth and family partners emphasised that engagement often extends to individuals transitioning out of paediatric services; therefore, we set an upper age limit of 24 years for inclusion within the scope of the review, while maintaining a youth focus by requiring that the mean age of engaged partners be 18 or under.

‘Engagement’ was defined in collaboration with our youth and family partners as an ongoing, two‐way exchange of information. Studies limited to one‐way communication, as described in *Inform* level activities on the International Association for Public Participation (IAP2) Spectrum [22], were excluded. Articles were excluded if they were unrelated to paediatric health or mental health institutions, lacked evidence of patient engagement, were not focused on health or mental healthcare, or did not address a paediatric population.

A full‐text review of studies was conducted as part of Level 2 screening, and any conflicts regarding the inclusion of studies at either level were resolved through team discussion. All reviewers involved in the evidence screening process convened virtually on a bi‐weekly basis to ensure consistency in how the inclusion criteria were applied and to critically reflect on the criteria for relevance and clarity. All revisions made to the inclusion/exclusion criteria during this dynamic and iterative process were systematically documented (Appendix B).

### 2.3 Data Extraction

To standardise the data extraction process, a data charting tool was developed in consultation with all study team members, including partners. The tool was pilot tested by four reviewers (B.A., F.B., I.D. and D.K.) independently on a random sample of 15 studies. Charting categories were revised iteratively whenever items were unclear or inconsistently extracted to ensure high consistency. Data were extracted for eight key categories across all included studies: (1) study characteristics, (2) study population characteristics, (3) patient/family/caregiver (PFC) partner characteristics, (4) engagement strategies, (5) methods of contribution, (6) reporting of patient and family engagement, and (7) outcomes/impact of engagement. Following data extraction, the data were cleaned by two team members (I.D. and L.L.). This process involved coding specific open‐text fields into organised categories in Excel. The codebook outlining and defining all categories extracted and included in the results section is provided in Supplemental File 1.

### 2.4 Data Analysis

**Figure 1:**
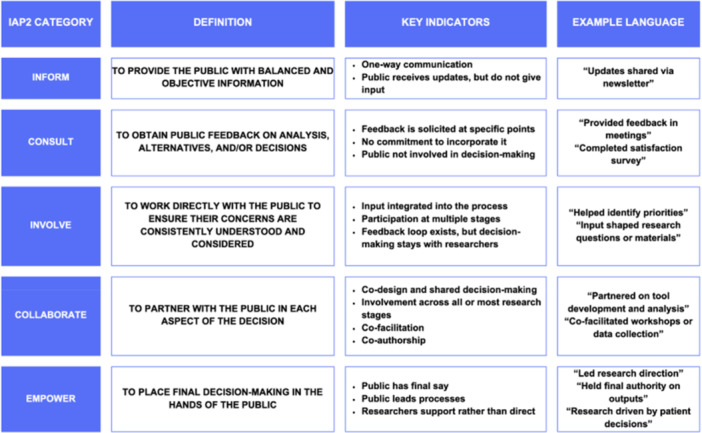
Engagement levels adapted from the IAP2 Spectrum of Public Participation. *The original IAP2 Spectrum comprises the *level, description* and *promise*; our adaptation focused on the *level* and added *key indicators* and *example language* to inform coding.

Extracted data was summarised descriptively, reviewed by three members of the research team (B.A., I.D. and D.K.) and categorised into one of three domains (clinical care, education or research). A uniform coding structure was applied using the IAP2 Spectrum of Public Participation (see Figure 1 for operational details on how engagement approaches were interpreted within this review). The IAP2 Spectrum of Public Participation was recommended by our youth and family partners, as it provides a theoretically grounded and widely utilised framework that is not explicitly oriented towards either paediatric or adult populations and encompasses engagement across different settings. Subsequently, frequencies and proportions were calculated for variables extracted under the categories of study characteristics (e.g., country of study), study population characteristics (e.g., participant condition type) and PFC partner characteristics (e.g., partner type and age range) to identify overarching trends. Open‐text data pertaining to engagement strategies, methods of contribution and the impacts of engagement were further analysed using line‐by‐line qualitative content analysis, in which three members of the research team (I.D., D.K. and L.N.) systematically reviewed each segment of text to identify and categorise recurring concepts [23]. Each reviewer independently analysed the data before meeting iteratively to reach consensus, ensuring consistency and shared interpretation across the team. In line with established scoping review methodology, a formal critical appraisal of included literature was not performed, considering the exploratory nature of scoping reviews and our study objective.

### 2.5 Patient and Public Involvement

This review was co‐designed and conducted alongside three youth and family partners with diverse lived experiences as patients or caregivers. One partner also served as co‐investigator on the research team. Each partner is listed as a co‐author and has been involved in research engagement to differing degrees, contributing unique perspectives and skills to the project. Table 1 outlines the breadth and impacts of partner involvement, which spanned all stages of the review.

**Table 1:** Impact of partnership on scoping review.

	Impact on research	Impact on researchers	Impact on partners
Study phase			
Protocol development	Suggested replacing the term ‘advisors’ with ‘partners’ throughout the review to reflect equal partnership	Encouraged researchers to think about the value and need for grey literature	Strengthened partners' understanding of the research methodology and prompted bi‐directional exchange of knowledge between partners and researchers
Selecting inclusion/exclusion criteria	Demonstrated the value of single consultations in engagement based on their own experiences and the types of resources to support activities (e.g., not only long‐term partnerships should be included)—expanded our inclusion criteria to include priority‐setting initiatives, consultations and so forth.	Encouraged researchers to track changes to inclusion criteria over time and how these evolved based on partner/team member feedback for transparency	Partners learned the importance of balancing study rigour and feasibility
Data screening	Advised on which studies were in/out of scope	Expanded researchers' understanding of the importance of engagement, regardless of the length or scope of involvement (e.g., illuminated the value in single consultations) and dissuaded PIs' bias towards longer‐term engagement initiatives	Partners gained confidence and developed competencies in evaluating scientific evidence and using Covidence, which helped them develop a greater appreciation for the vast and diverse body of literature
	Helped clarify specific articles that should be included or excluded based on partner involvement—e.g., participants vs. active collaborators; relevance of community‐based research to paediatric institutions		
	Helped determine order of exclusion in screening list (e.g., what comes up first when reading an article?)		
Data extraction	Advised on language used to describe those being engaged (‘those being engaged’ selected in place of advisors or partners in included studies, given variability in their roles/tasks)	Advised the research team to include new data extraction categories that had not been previously considered based on their lived experience, including specifying the language used to define those being engaged	Partners felt that they were recognised as experts in their own right as they led the charge to develop the initial set of extraction categories in a way that aligned with their lived experiences and self‐identified priorities
	Developed an initial set of categories for extraction, which were then refined by the research team. Made concrete changes to the data extraction tool, including categorising the impact of engagement on partners, researchers, research and adding new headings (language used to describe advisors, whether participants/advisors are the same)	Made evident to the PI the power of language in engagement, the importance of clarifying roles at the outset of partnerships, and the need for transparent definitions in the literature	
Data interpretation	Assisted in refining the results section, assisting with which key findings should be included or excluded. Improved clarity, flow and grammar throughout the manuscript	Highlighted the importance of avoiding tokenism by emphasising meaningful involvement in data interpretation, writing and editing rather than limiting contributions to purely quantitative tasks	Empowered partners to ensure that their voices and lived experiences were authentically represented in the overarching narrative of the review, including gaps and future directions
Knowledge translation	Conceptualised and developed a figure to visualise the core findings of the manuscript	Challenged researchers to consider the importance of visually appealing, accessible methods for conveying information about study findings to broad audiences	Being actively involved in knowledge translation instilled partners with a strong sense of ownership, especially seeing their efforts being formally recognised through verbal acknowledgements, remuneration and co‐authorship
	Encouraged inclusion of the complexity of categorising what constitutes ‘engagement’ throughout the manuscript		
	Identified conferences and events that would be good venues for presentation		
	Assisted in identifying appropriate journals for manuscript submission by reviewing scope, audience and relevance to patient‐partnered research		
	Provided constructive feedback on conference posters, including clarity of messaging, accessibility of language and visual presentation		

The establishment of the youth and family partner group was guided by the Canadian Institutes of Health Research (CIHR) Strategy for Patient‐Oriented Research (SPOR) framework [1] and the McCain Model [24], both of which emphasise meaningful engagement of patients and families in health research, service design and decision‐making. Recruitment of partners occurred through the [*department name*] at the [*hospital name*], with all members having a pre‐existing relationship with the hospital through prior involvement in various initiatives.

Engagement with partners occurred through bi‐weekly virtual meetings, scheduled using virtual polls to ensure that they aligned with everyone's availability. Meetings were recorded for those unable to attend, with one‐on‐one meetings additionally offered to ensure all members had the opportunity to provide input. Each meeting included icebreakers to foster rapport, as well as project updates and discussions on various aspects of the review. Training on research methods, such as Covidence screening procedures and grey literature searches, was offered. Partners were also invited to complete a *Methods of Contribution* form (Appendix C), empowering them to volunteer to participate in phases of the review that aligned with their individual preferences and capacities for involvement. A *Terms of Reference* document was shared with each partner, clearly outlining the research goals, roles, responsibilities and timelines. All partners were compensated at a rate of $35 per hour for their involvement in the project, in accordance with the CIHR compensation guidelines [25]. An additional $15 per hour was also made available for those requiring child/elder care to support their participation in the meetings.

## 3 Results

**Figure 2:**
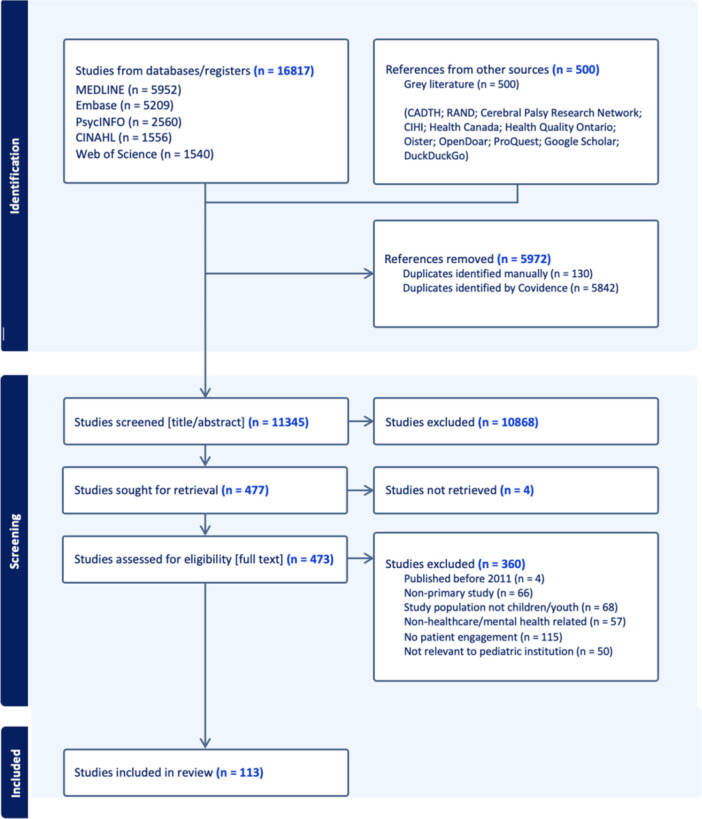
PRISMA flow diagram.

**Figure 3:**
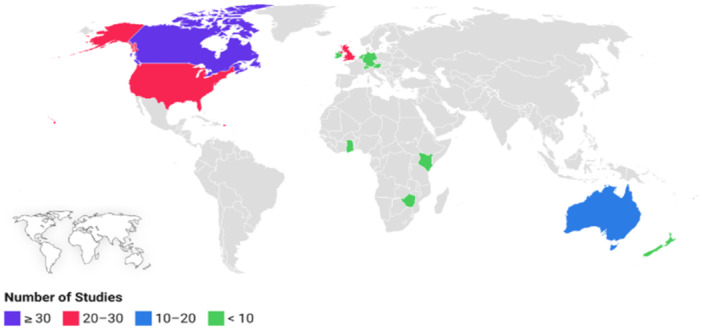
Geographic distribution of included studies.

**Figure 4:**
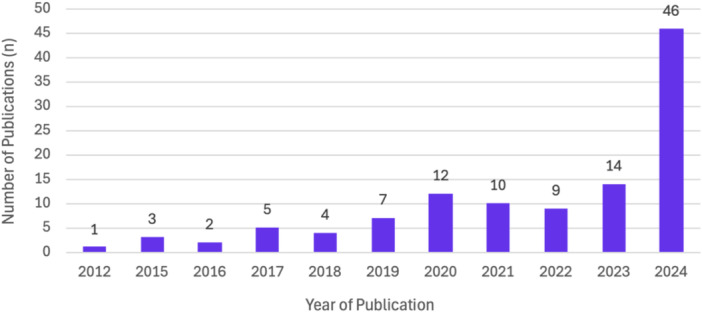
Distribution of included studies by year.

The initial database search of the published literature identified 16,817 records. After excluding duplicates, 10,846 unique studies were screened and a total of 113 articles met eligibility criteria (Figure 2). The citations and key characteristics of the included studies are provided in Supplemental File 2. Most studies included in our review were conducted in Canada and the United Kingdom (Figure 3). A notable trend across the included literature is the increasing prominence of paediatric engagement, with most articles published in 2024 (Figure 4).

### 3.1 Study Characteristics

Of the articles reviewed, 50% (*n* = 56) addressed engagement in research, 47% (*n* = 53) in clinical care and only 3% (*n* = 4) in education. The limited number of articles in the education domain was largely attributable to the exclusion of studies that targeted general healthcare professional training rather than paediatric‐focused educational initiatives. Across all domains, physical health conditions were by far the most common focus (*n* = 76), with a relatively even distribution across domains (clinical care = 71.7%; education = 50%; research = 64.3%). Neurodevelopmental and developmental conditions, by contrast, were concentrated in the research domain, representing 70% of all neurodevelopmental studies and 64.7% of all developmental studies. In the education domain, half of the studies did not specify a condition type (50%), while the remaining half focused on physical health conditions (50%). Although study settings varied, the majority took place in clinical care settings (i.e., hospitals) (*n* = 64) and universities (*n* = 43). Across domains, government agencies appeared least frequently (n = 5), and the majority of these studies (80%) were situated within the research domain. Community‐based settings were also uncommon (*n* = 16), with most occurring within the research domain (62.5%) and a smaller proportion within the clinical care domain (31.3%). By contrast, clinical care settings were represented relatively evenly across domains, appearing in 58.5% of clinical care studies, 75% of education studies and 53.6% of research studies. The education domain had no studies conducted within research centres or government settings. A more detailed breakdown of study characteristics by domain is presented in Table 2.

**Table 2:** Study characteristics.

	Clinical care (53)	Education (4)	Research (56)	Total (113)
**Setting** *				
Clinical care University Research centre Community‐based Government	31 (58.5%) 19 (35.8%) 6 (11.3%) 5 (9.4%) 1 (1.9%)	3 (75%) 1 (25%) 0 (0%) 1 (25%) 0 (0%)	30 (53.6%) 23 (41.1%) 11 (19.6%) 10 (17.9%) 4 (7.1%)	64 (56.6%) 43 (38.1%) 17 (15%) 16 (14.2%) 5 (4.4%)
**Condition type** *				
Mental Physical Developmental Neurodevelopmental Not specified	11 (20.7%) 38 (71.7%) 6 (11.3%) 3 (5.7%) 1 (1.9%)	0 (0%) 2 (50%) 0 (0%) 0 (0%) 2 (50%)	8 (14.3%) 36 (64.3%) 11 (19.6%) 7 (12.5%) 8 (14.3%)	19 (16.8%) 76 (67.3%) 17 (15%) 10 (8.8%) 11 (9.7%)
**Study methodology**				
Qualitative Mixed methods Multi method Delphi JLA Reflective Randomised controlled trial QI Other	20 (37.7%) 8 (15.1%) 12 (22.6%) 2 (3.8%) 0 (0%) 1 (1.9%) 3 (5.7%) 5 (9.4%) 2 (3.8%)	0 (0%) 2 (50%) 1 (25%) 0 (0%) 0 (0%) 0 (0%) 0 (0%) 0 (0%) 1 (25%)	21 (37.5%) 7 (12.5%) 6 (10.7%) 3 (5.4%) 9 (16.1%) 2 (3.6%) 0 (0%) 0 (0%) 8 (14.3%)	41 (36.3%) 17 (15%) 19 (16.8%) 5 (4.4%) 9 (8%) 3 (2.7%) 3 (2.7%) 5 (4.4%) 11 (9.7%)

* Multiple categories may be selected per study.

### 3.2 Characteristics of Those Being Engaged

The level of detail about who was engaged varied considerably across studies, often leaving critical information about characteristics incomplete or inconsistently reported. Within the clinical care domain, studies most frequently engaged caregivers exclusively (32.1%), whereas in the research domain, engagement most often involved both patients and caregivers (33.9%). In the education domain, engagement was evenly divided between studies involving only patients (50%) and only caregivers (50%). No studies engaged caregivers and family members together as a sole, standalone group without patient involvement. Demographic characteristics were reported in 39% of studies, with most including information only on sex/gender (31.9%) and race/ethnicity (19.5%). Geographic location (9.7%), educational attainment (7.1%), socio‐economic status (2.7%) and sexual identity (2.7%) were also reported.

When examining partner recruitment methods, purposeful or targeted sampling approaches were the most common (47.8%), appearing most frequently in the clinical care domain (57.4%) and also extensively in the research domain (42.6%). Education studies relied exclusively on existing networks (*n* = 3) [11, 26, 27], with one study not reporting its recruitment method. Notably, 84.1% of studies did not incorporate any EDI considerations into recruitment. The education domain reported no EDI‐informed recruitment strategies, and non‐reporting was similarly high in both clinical care (84.9%) and research (82.1%). Among the few studies that did apply EDI principles to partner recruitment, broad outreach intended to engage an equitable and inclusive range of individuals was most common (5.3%) [28, 29, 30] and appeared primarily in the clinical care domain (66.7%), whereas targeted outreach explicitly focused on marginalised groups (1.8%) [31, 32] and other specific inclusion approaches were rarely used across domains (Table 3).

**Table 3:** Characteristics of those being engaged.

	Clinical care (53)	Education (4)	Research (56)	Total (113)
**Partner type** *				
Patient only	11 (20.8%)	2 (50%)	15 (26.8%)	28 (24.8%)
Family only	4 (7.5%)	0 (0%)	2 (3.6%)	6 (5.3%)
Caregiver only	17 (32.1%)	2 (50%)	11 (19.6%)	30 (26.5%)
Patient + family	1 (1.9%)	0 (0%)	4 (7.1%)	5 (4.4%)
Patient + caregiver	16 (30.2%)	0 (0%)	19 (33.9%)	35 (31%)
Caregiver + family	0 (0%)	0 (0%)	0 (0%)	0 (0%)
Patient + family + caregiver	4 (7.5%)	0 (0%)	5 (8.9%)	9 (8%)
**Number of PFC partners**				
1–5 6–10 11–19 20–99 100+ Not reported	8 (15.1%) 17 (32.1%) 7 (13.2%) 9 (17%) 1 (1.9%) 11 (20.8%)	2 (50%) 1 (25%) 0 (0%) 0 (0%) 0 (0%) 1 (25%)	18 (32.1%) 8 (14.3%) 12 (21.4%) 6 (10.7%) 3 (5.4%) 9 (16.1%)	28 (24.8%) 26 (23%) 19 (16.8%) 15 (13.3%) 4 (3.5%) 21 (18.6%)
**Top 10 languages used** *				
Parent(s) Partner(s) Patient(s) Participant(s) Young people Co‐Researcher(s) Advisor(s) Caregiver(s) Steering Group/Committee Advisory Group/Council/Board	11 (20.8%) 4 (7.5%) 2 (3.8%) 9 (17%) 3 (5.7%) 1 (1.9%) 8 (15.1%) 1 (1.9%) 1 (1.9%) 5 (9.4%)	0 (0%) 1 (25%) 1 (25%) 0 (0%) 0 (0%) 0 (0%) 0 (0%) 0 (0%) 2 (50%) 0 (0%)	15 (26.8%) 11 (19.6%) 5 (8.9%) 0 (0%) 4 (7.1%) 4 (7.1%) 2 (3.6%) 4 (7.1%) 5 (8.9%) 6 (%)	26 (23%) 16 (14.2%) 8 (7.1%) 9 (8%) 7 (6.2%) 5 (4.4%) 10 (8.8%) 5 (4.4%) 8 (7.1%0 11 (9.7%)
**Recruitment methods** *				
Existing networks Purposeful/Targeted Open call Snowball Convenience Not reported	26 (49.1%) 31 (58.5%) 7 (13.2%) 6 (11.3%) 8 (15.1%) 11 (20.8%)	3 (75%) 0 (0%) 0 (0%) 0 (0%) 0 (0%) 1 (25%)	22 (39.3%) 23 (41.1%) 10 (17.9%) 6 (10.7%) 4 (7.1%) 9 (16.1%)	51 (45.1%) 54 (47.8%) 17 (15%) 12 (10.6%) 12 (10.6%) 21 (18.6%)
**EDI considered in recruitment?** *				
Geographic inclusion Literacy inclusion Language inclusion Age inclusion Targeted outreach (marginalised) Representation focused Sex and gender inclusion Cultural/Racial/Ethnic inclusion System/Service access inclusion Flexible/Inclusive criteria Condition variety/Severity inclusion Patient‐oriented research experience Not reported	0 (0%) 0 (0%) 0 (0%) 1 (1.9%) 0 (0%) 4 (7.5%) 1 (1.9%) 2 (3.8%) 1 (1.9%) 1 (1.9%) 3 (5.7%) 0 (0%) 45 (84.9%)	0 (0%) 0 (0%) 0 (0%) 0 (0%) 0 (0%) 0 (0%) 0 (0%) 0 (0%) 0 (0%) 0 (0%) 0 (0%) 0 (0%) 4 (100%)	4 (7.1%) 1 (1.8%) 2 (3.6%) 4 (7.1%) 2 (3.6%) 2 (3.6%) 4 (7.1%) 3 (5.4%) 1 (1.8%) 1 (1.8%) 2 (3.6%) 2 (3.6%) 46 (82.1%)	4 (3.5%) 1 (0.9%) 2 (1.8%) 5 (4.4%) 2 (1.8%) 6 (5.3%) 5 (4.4%) 5 (4.4%) 2 (1.8%) 2 (1.8%) 5 (4.4%) 2 (1.8%) 95 (84.1%)

* Multiple categories may be selected per study.

The language used to describe engaged individuals varied widely across domains. The clinical care domain was the only setting to use the term *participants* (*n* = 9), even though the research domain made the greatest use of JLA priority‐setting partnerships and Delphi processes, which are typically described in the literature as involving *participants*. Both the research and clinical care domains most commonly referred to engaged individuals as *parents* (clinical care = 20.8%; research = 26.8%) [33, 34, 35], whereas the education domain primarily used the term *steering group/committee* (50%) [11, 36].

#### 3.2.1 Engagement Methods and Roles

Across all three domains, studies most frequently engaged between 1 and 10 individuals as partners (clinical care = 47.2%; education = 75%; research = 46.4%). However, there were distinctions regarding the number of partners engaged based on the specific methods employed in each domain. For instance, studies within the research domain tended to engage a larger number of partners, with three studies involving more than 100 individuals [31, 34, 37], compared to one in the clinical domain [38] and none in the education domain. Methods such as Delphi processes and JLA priority‐setting partnerships (both commonly situated within the research domain) typically engaged a higher number of individuals, given that their multi‐round consultation format draws on input from large groups of different stakeholders. We identified nine studies that used JLA methods for engagement [31, 39, 40, 41, 42, 43, 44, 45, 46], all within the research domain. Three out of five Delphi studies (60%) were also applied within research contexts [37, 47, 48]. All five quality improvement (QI) projects occurred within the clinical care domain [49, 50, 51, 52, 53]. Generally, studies employing more collaborative approaches (e.g., engaging partners via co‐creation activities, workshops, testing and evaluation methods) were more prevalent in the clinical domain and engaged mid‐sized groups (6–10 partners) [54, 55], typically through advisory groups. Outside of larger multi‐round priority setting initiatives, studies in the education and research domains engaged a much smaller number of partners overall, typically ranging from 1 to 5 individuals [20, 27]. A higher proportion of studies in the clinical care (20.8%) and education (25%) domains did not report the number of partners engaged in comparison to the research domain (16.1%).

As outlined in Table 4, a range of engagement methods were identified across the included studies. The most prevalent approaches overall were meetings (*n* = 44), workshops (*n* = 39), co‐creation activities (*n* = 38) and advisory groups (*n* = 30); however, domain‐level distinctions were observed. Co‐creation activities were employed heavily within the clinical care domain (49.1%) [56, 57, 58], relative to education (25%) [26] and research (19.6%) [34, 59, 60], suggesting a greater emphasis on collaborative development in clinical contexts. Conversely, committees or governance‐oriented meetings were most frequently used in the education domain (75%) [11, 27, 36], consistent with the terminology commonly applied to describe partners in this setting. Meetings were used most often in the research domain (38.9%) [41, 61], followed closely by clinical care (35.8%) [52, 62]. The clinical care domain also exhibited the greatest methodological breadth, with each engagement method appearing in at least three studies. Reflecting the engagement methods, co‐developing tools and materials was the most common role for engaged individuals in the clinical care domain (43.4%) [63, 64, 65], while providing feedback and review was the most frequently held role in both the education (100%) [11, 26, 27, 36] and research domains (33.9%) [61, 66, 67]. Remaining roles/responsibilities such as knowledge translation [68, 69] and study conceptualisation [49, 70] are outlined in Table 4.

**Table 4:** Engagement methods and roles by domain.

	Clinical care (53)	Education (4)	Research (56)	Total (113)
**Engagement method** *				
Workshops Focus groups Interviews Meetings Surveys/Questionnaires Advisory groups Co‐creation activities Testing and evaluation Asynchronous collaboration Consensus and priority‐setting methods Steering committees/Governance meetings Document review and written feedback	19 (35.8%) 16 (30.2%) 13 (24.5%) 19 (35.8%) 4 (7.5%) 12 (22.6%) 26 (49.1%) 5 (9.4%) 10 (18.9%) 3 (5.7%) 3 (5.7%) 5 (9.4%)	2 (50%) 0 (0%) 0 (0%) 1 (25%) 0 (0%) 0 (0%) 1 (25%) 1 (25%) 1 (25%) 2 (50%) 3 (75%) 0 (0%)	18 (32.1%) 11 (19.6%) 7 (12.5%) 24 (42.9%) 6 (10.7%) 18 (32.1%) 11 (19.6%) 1 (1.8%) 13 (23.2%) 12 (21.4%) 7 (12.5%) 6 (10.7%)	39 (34.5%) 27 (23.9%) 20 (17.7%) 44 (38.9%) 10 (8.8%) 30 (26.5%) 38 (33.6%) 7 (6.2%) 24 (21.2%) 17 (15%) 13 (11.5%) 11 (9.7%)
**Roles and responsibilities** *				
Co‐develop tools/materials Feedback and Review Knowledge translation and dissemination Intervention development and refinement Data analysis and interpretation Study conceptualisation and protocol development Co‐facilitation Topic/Priority setting Data collection and participation Recruitment and outreach Sharing lived experiences Governance and decision‐making	23 (43.4%) 21 (39.6%) 14 (26.4%) 15 (28.3%) 12 (22.6%) 12 (22.6%) 8 (15.1%) 11 (20.8%) 5 (9.4%) 5 (9.4%) 9 (17%) 7 (13.2%)	2 (50%) 4 (100%) 0 (0%) 0 (0%) 1 (25%) 0 (0%) 1 (25%) 0 (0%) 1 (25%) 0 (0%) 0 (0%) 0 (0%)	15 (26.8%) 19 (33.9%) 16 (28.6%) 17 (30.4%) 17 (30.4%) 14 (25%) 15 (26.8%) 14 (25%) 4 (7.1%) 6 (10.7%) 6 (10.7%) 6 (10.7%)	40 (35.4%) 44 (38.9%) 30 (26.5%) 32 (28.3%) 30 (26.5%) 26 (23%) 24 (21.2%) 25 (22.1%) 10 (8.8%) 11 (9.7%) 15 (13.3%) 13 (11.5%)
**EDI considered in engagement methods?** *				
Flexible scheduling Appropriate venues Cultural responsiveness Remote and multi‐modal engagement Psychological safety and safe spaces Accessible communication and inclusive formats Practical and logistical supports Variations in group structuring Not reported	2 (3.8%) 1 (1.9%) 8 (15.1%) 5 (9.4%) 1 (1.9%) 5 (9.4%) 5 (9.4%) 1 (1.9%) 37 (69.8%)	0 (0%) 0 (0%) 0 (0%) 0 (0%) 1 (25%) 0 (0%) 0 (0%) 0 (0%) 3 (75%)	5 (8.9%) 2 (3.6%) 3 (5.4%) 8 (14.3%) 13 (23.2%) 8 (14.3%) 3 (5.4%) 4 (7.1%) 34 (60.7%)	7 (6.2%) 3 (2.7%) 11 (9.7%) 13 (11.5%) 15 (13.3%) 13 (11.5%) 8 (7.1%) 5 (4.4%) 74 (65.5%)
**Training provided?**				
Yes No	7 (13.2%) 46 (87.8%)	0 (0%) 4 (100%)	13 (23.3%) 43 (76.8%)	20 (17.7%) 93 (82.3%)
**Recognition provided?** *				
Remuneration Co‐authorship Education credits Written acknowledgement Not reported	17 (32.1%) 11 (20.8%) 1 (1.9%) 7 (13.2%) 24 (45.3%)	1 (25%) 1 (25%) 0 (0%) 1 (25%) 2 (50%)	17 (30.4%) 16 (28.6%) 1 (1.8%) 9 (16.1%) 23 (41.1%)	35 (31%) 28 (24.8%) 2 (1.8%) 17 (15%) 49 (43.4%)

* Multiple categories may be selected per study.

The three most common forms of recognition for partners were consistent across all three domains. No form of recognition was most frequently cited in this category (clinical care = 45.3%; education = 50%; research = 41.1%), followed by providing remuneration (clinical care = 32.1%; education = 25%; research = 30.4%) [26, 48, 71] and co‐authorship (clinical care = 20.8%; education = 25%; research = 28.6%) [27, 57, 72]. We aimed to capture the specific objectives of engagement within each study; however, extraction revealed that most articles reported only the overarching research objectives rather than the distinct purpose or goals of engagement itself. For research objectives, however, the most common aim across all domains was the collaborative creation or refinement of tools (46%) [73, 74, 75, 76], which may reflect the underlying purpose of engagement itself. Other research objectives such as engagement process evaluation (31.9%) [77, 78], experience exploration (26.5%) [79, 80] and evaluation and usability testing (23.9%) [68, 81] were also common. Only 20 studies reported providing training to partners, and most of these (65%) took place in the research domain [67, 82]. This is consistent with the type of training most commonly reported, which was training focused on research‐related skills and knowledge (e.g., research methods, ethics, data collection, analysis, qualitative coding and academic writing) (*n* = 10) [70, 83]. Other forms of training, such as facilitation training (*n* = 6) [84, 85] and project/process orientation (*n* = 3) [39, 86, 87], were less frequently reported. With respect to the duration of engagement, reported timeframes ranged from as little as 1.5 h [88] to as long as 6 years [89].

Only 39 studies described a commitment to EDI principles in their engagement methods. When present, researchers most commonly addressed efforts to promote psychological safety and create safe spaces for engagement (*n* = 15) [78, 89], although these efforts were largely concentrated within the research domain (80%) [90]. Overall, the research domain demonstrated the greatest adoption of EDI‐oriented strategies, including variation in group structuring (80%) [90, 91], accessible communication and inclusive formats (61.5%) [71, 92], flexible scheduling (71.4%) [53, 89] and the provision of appropriate venues (66.7%) [93, 94]. In contrast, the clinical care domain most frequently employed culturally responsive approaches (72.3%) [95, 96]. Reporting in the education domain was limited; 75% of studies provided no information on EDI‐related methods, with only one study describing efforts to promote psychological safety [26].

### 3.3 Engagement Reporting and Evaluation

The impacts of engagement on either the research itself, researchers or partners were reported in a total of 92 studies (Table 5). Impacts on research were the most frequently described, appearing in 44.2% of studies. Interestingly, the research domain was the least likely to report on this category of impact, whereas the education domain did so in 75% of its studies [11, 26, 27]. Across all domains, the most commonly reported research‐related impact was increased relevance to end users (clinical care = 24.5%; education = 25%; research = 30.4%) [27, 65, 77]. Domain‐specific differences also emerged; for example, clinical care studies more often reported broadened accessibility of research outputs (55.6%) [55, 71], a finding that aligns with the predominance of co‐creation approaches in that domain. The impact of engagement on partners was described in only 30 studies and was minimal across all domains (clinical care = 24.5%; education = 25%; research = 26.6%). When reported, capacity development was described most frequently (i.e., partners gained knowledge, new skills or experienced professional development) [70, 83, 97] in the research domain. In the clinical domain, partners most often reported developing an appreciation for authentic engagement (11.3%) [53, 98], which was less often reported in research studies (3.6%) [47, 99] and not at all in education (0%). Within the education domain, impacts on partners were only reported in one study [26]. Only 12 studies reported the impacts of engagement on the research team, none of which were situated within the education domain. Reporting was similarly limited in the clinical care and research domains (clinical care = 9.9%; research = 12.5%). Among the studies that did describe such impacts, the most common was that engagement prompted personal reflection of values and research practices within the research team (clinical care = 37.5%; research = 62.5%) [78, 100, 101, 102].

**Table 5:** Engagement impacts and tools by domain.

	Clinical care (53)	Education (4)	Research (56)	Total (113)
**Engagement category** *				
Inform Consult Involve Collaborate Empower	— 10 (18.9%) 27 (50.9%) 26 (49.1%) 1 (1.9%)	— 0 (0%) 0 (0%) 4 (100%) 0 (0%)	— 6 (10.7%) 28 (50%) 28 (50%) 5 (8.9%)	— 16 (14.2%) 55 (48.7%) 58 (51.3%) 6 (5.3%)
**Tools used to report engagement?**				
GRIPP2 REPRISE Not reported	1 (2%) 0 (0%) 52 (98%)	2 (50%) 0 (0%) 2 (50%)	11 (19.6%) 1 (1.8%) 44 (78.6%)	14 (12.4%) 1 (0.9%) 98 (86.7%)
**Evaluation of engagement**				
PPEET PEIRS Study‐specific tools Not reported	1 (2%) 1 (2%) 4 (8%) 47 (89%)	1 (25%) 0 (0%) 0 (0%) 3 (75%)	2 (3.6%) 0 (0%) 7 (12.5%) 47 (83.9%)	4 (3.5%) 1 (0.9%) 11 (9.7%) 97 (85.8%)
**Impact of engagement reported on research?** *				
Increased relevance to end users Outputs grounded in lived experiences Increased methodological rigour Broadened accessibility of research outputs Impacted recruitment and reach Not reported	13 (24.5%) 3 (5.7%) 5 (9.4%) 10 (18.9%) 4 (7.5%) 29 (54.7%)	1 (25%) 0 (0%) 0 (0%) 1 (25%) 1 (25%) 1 (25%)	17 (30.4%) 9 (16.1%) 14 (25%) 7 (12.5%) 6 (10.7%) 33 (58.9%)	31 (27.4%) 12 (10.6%) 19 (16.8%) 18 (15.9%) 11 (9.7%) 63 (55.8%)
**Impact of engagement reported on partners?** *				
Felt valued Felt empowered Capacity development Developed an appreciation of authentic engagement Felt contributions meaningfully impacted research Felt comfortable to express perspectives Identified/Strengthened connections within community Not reported	1 (1.9%) 3 (5.7%) 4 (7.5%) 6 (11.3%) 3 (5.7%) 4 (7.5%) 2 (3.8%) 40 (75.5%)	0 (0%) 0 (0%) 0 (0%) 0 (0%) 1 (25%) 1 (25%) 0 (0%) 3 (75%)	5 (8.9%) 5 (8.9%) 7 (12.5%) 2 (3.6%) 1 (1.8%) 4 (7.1%) 1 (1.8%) 40 (71.4%)	6 (5.3%) 8 (7.1%) 11 (9.7%) 8 (7.1%) 5 (4.4%) 9 (8%) 3 (2.7%) 83 (73.5%)
**Impact of engagement reported on researchers?** *				
Built engagement capacity/Proficiency Understood partner's perspectives Inspired personal reflection of values and research process Not reported	1 (1.9%) 2 (3.8%) 3 (5.7%) 48 (90.1%)	0 (0%) 0 (0%) 0 (0%) 4 (100%)	3 (5.4%) 1 (1.8%) 5 (8.9%) 49 (87.5%)	4 (3.5%) 3 (2.7%) 8 (7.1%) 101 (89.4%)

* Multiple categories may be selected per study.

Each study was assessed using the IAP2 Spectrum of Public Participation to determine its level of engagement, ranging from *Inform* to *Empower* (Figure 1). Only four studies [26, 27, 54, 81] explicitly reported their level of engagement, all of which were within the clinical and education domains, with the remainder interpreted and classified by our team. Of the four articles identified within the education domain, all met the criteria for the *Collaborate* level of engagement [11, 26, 27, 36] with none dually classified under any other level. In the research domain, studies were evenly divided between the *Involve* and *Collaborate* levels (*n* = 28 each) [67, 80, 85, 103] and included the majority of *Empower* studies (5 of 6) [69, 87, 97, 102] as well as the fewest *Consult* studies (6 of 16) [28, 77, 79, 104, 105, 106]. Within the clinical domain, most studies were classified at the *Involve* level (*n* = 27) [92, 107], followed closely by *Collaborate* (*n* = 26) [74, 108] with fewer at *Consult* (*n* = 10) [35, 88] and only one at *Empower* (*n* = 1) [54]. Across all domains, no studies were categorised at the *Inform* level.

Reporting of the engagement process using formal tools or guidelines was uncommon across domains, with only 15 studies in this review doing so. However, there were some distinctions in how often reporting tools were used within specific domains. Of the articles that used formal tools, the majority (80%) were within the research domain. The Guidance for Reporting Involvement of Patients and the Public (GRIPP2) checklist [109] was the primary reporting tool used (*n* = 14) [73, 77], with REPRISE [110] appearing only once [31], also within the research domain. Notably, more studies in the education domain reported using formal tools (50%) [26, 27] than those in the clinical domain (2%) [55]. Findings were similar when examining the formal evaluation of the engagement process itself. Both the research and clinical domains showed an equal absence (*n* = 47 studies each) of any kind of formal evaluation. Only one of the four education studies reported conducting a formal evaluation [26]. Among studies that *did* evaluate engagement, the tools used included the Patient Engagement in Research Scale (PEIRS) [111], the Public and Patient Engagement Evaluation Tool (PPEET) [112] and study‐specific instruments such as questionnaires or interviews [53, 93, 113]. Evaluations were conducted either by members of the research team (*n* = 12) [55, 89] or external staff (*n* = 5) [67, 114], while patient partners (*n* = 2) [84, 87] and family partners (*n* = 2) [115, 116] were involved in this process significantly less often.

## 4 Discussion

This review identified the commonalities and distinctions in paediatric patient and family engagement across clinical care, research and education, providing a foundation for understanding where practices align, where they diverge and where persistent gaps remain. It reported on patterns in the engagement methods applied, the roles and responsibilities of partners, the impacts of engagement, and how engagement was evaluated across domains. The review showed that publications in this field have increased over time, with most studies focusing on clinical care and research rather than education, similar to prior research in paediatric patient engagement [117].

The review revealed commonalities across domains that demonstrate shared commitments to meaningful engagement. More than half of the studies provided some form of recognition to their partners, such as remuneration or co‐authorship, indicating that partners' time and expertise were being valued within these engagement processes. Small engagement groups (between 1 and 10 individuals) and co‐development and feedback roles were common across domains. Interestingly, the engagement methods observed most frequently in this review (i.e., workshops, meetings and co‐creation activities) differ from a prior scoping review, which found that interviews, focus groups and draw and write techniques were the most common approaches for engaging paediatric patients in clinical care and research [117]. This could be a result of how ‘engagement’ was defined in our study and the fact that the current review included studies which involved caregivers and family members as partners in addition to paediatric patients. Additionally, engagement in most of the included studies fell at either the *Involve* or *Collaborate* level on the IAP2 Spectrum. The small number of articles that involved partners in activities commensurate with the *Empower* level aligns with Maini et al.'s [118] recent call for more patient‐led initiatives to amplify the perspectives of those with lived experience in health research. The findings of this review reinforce the need for such initiatives within clinical care and education contexts as well. In addition, our results suggest conceptual overlap between family‐centred care and the engagement principles examined, including partnership and collaboration [119]. However, the relationship between these concepts is not consistently articulated in the literature, underscoring the need for further work to clarify how family‐centred care and patient engagement intersect within paediatric institutions.

The scoping review identified similarities regarding the limited reporting of the impact of engagement across all three domains. When present, most studies described their impact on the research itself, and less commonly, on researchers and partners. Nearly three‐quarters of studies did not describe what partners gained from their involvement, representing a substantial gap in the evidence base. Assessing partner‐level impacts is essential for understanding what fosters truly meaningful experiences and sustained engagement for partners, as well as for ensuring they are valued and supported consistently across all engagement settings. The absence of such reporting may stem from limitations in engagement evaluations to assess the partner experience and personal impact; only 15 studies formally reported evaluations of their engagement processes. This finding points to the need for templates or guidance regarding how to report on impact within paediatrics, regardless of the context of engagement [120].

A final area of convergence across domains was the limited incorporation of EDI principles in both recruitment of partners and engagement practices. Studies incorporated EDI considerations into their engagement methods at nearly double the rate observed in their recruitment strategies. While the incorporation of culturally responsive practices (primarily observed in clinical care) and the development of psychologically safe environments for engagement (frequently applied in education and research) were both promising strategies employed, an opportunity for more consistent application and reporting of these approaches exists across domains. EDI‐informed recruitment methods, which require intentional and proactive efforts to include individuals from marginalised groups in engagement activities, were rarely reported in the studies included in this review. This issue has been highlighted in prior research focused primarily on youth engagement in research [102]; however, the present review confirms that similar gaps exist in clinical care and education.

In addition to commonalities, several important distinctions across domains were observed. One of the key differences was the language used to describe engaged individuals. The clinical care domain was the only area in which engaged individuals were described as *participants*, whereas the research domain most frequently used the terms *steering committee* or *steering group*. The education domain relied primarily on *partners*, *patients* and *steering committee/group*. This lack of standardisation has been described in prior studies focused on patient engagement [117, 121, 122, 123]. Without shared terminology, it becomes difficult to compare engagement practices, assess the extent of involvement, or translate successful approaches across settings.

Differences across domains were further reflected in the composition of engaged individuals and their roles and responsibilities. Education studies engaged either patients or caregivers exclusively and did not include other family members or mixed groups, while clinical care studies most often engaged caregivers. Prior reviews similarly note that caregiver involvement is often more feasible than directly engaging paediatric patients due to developmental, ethical and logistical constraints, including consent and assent considerations, variability in children's capacity to participate, and the practical challenges of involving families with complex schedules or competing demands [15, 124]. Partner roles in clinical care most often took the form of co‐developing materials, while feedback and review were mostly commonly reported in the education and research domains. Governance and decision‐making roles for partners were observed in clinical care and research settings, but not in education. Given that most studies did not explicitly state the goal(s) of engagement, we could not ascertain how these goals aligned (or not) with the engagement methods employed. Future research studies that engage paediatric patients and family members as partners across domains should aim to consistently describe the purpose of engagement to enhance transparency and accountability, including in how roles and responsibilities are determined.

Reporting of engagement processes varied substantially across domains. The research domain was the only setting in which reporting tools such as GRIPP2 [109] or REPRISE [110] were employed, although their use was infrequent. Limited reporting reduces transparency for partners and collaborators, impedes replication of effective practices, and constrains the field's capacity to identify what elements of engagement contribute to meaningful outcomes. Without clear documentation, opportunities to refine methods, build cumulative knowledge and develop evidence‐informed guidelines for high‐quality engagement are significantly diminished.

### 4.1 Implications for Practice

Several frameworks exist for supporting engagement within specific domains [22, 24, 125, 126]; however, few models exist which guide cross‐domain implementation [124]. Canada's Strategy for Patient‐Oriented Research has advanced patient engagement within the research domain by emphasising governance, capacity‐building and inclusion and by establishing SUPPORT Units across provinces [127]. However, its guidance remains largely confined to research and does not extend to clinical or educational settings. Similarly, GRIPP2 [109] and REPRISE [110] provide reporting guidance for research and priority‐setting exercises, but offer little applicability beyond these contexts. Other existing instruments such as the PEIRS [111] and PPEET [112] are not designed specifically for paediatrics or family engagement, nor do they evaluate engagement across domains, further constraining the ability to capture impacts. Even widely cited models such as the IAP2 Spectrum [22] and Hart's Ladder [128] illustrate levels of participation but provide limited direction on how to embed and sustain engagement in paediatric institutions where clinical care, education and research co‐occur.

The evidence base in this review was weighted towards the research and clinical domains; thus, the patterns identified may reflect clinical and research‐specific norms more than shared cross‐domain principles. Nonetheless, this review identified engagement practices that cut across settings (e.g., reporting impacts and recognising partners) as well as domain‐specific differences (e.g., the number of partners involved and the methods used) that attend to the unique goals and contexts of engagement. Documenting these similarities and differences creates opportunities for cross‐domain learning and the potential for consistent and equitable engagement experiences for paediatric patients and family partners, regardless of the context of engagement. Indeed, the evolving conceptual and educational literature on family‐centred care emphasises the value of involving children and families in healthcare decision‐making [119, 129, 130], reinforcing the need for greater cross‐sectoral collaboration in engagement practice and scholarship. While some domain‐specific variation is appropriate given differing mandates and ethical considerations, a shared foundation would support greater coherence and transferability, enabling each setting to build on common structures without losing necessary nuance. In practice, such infrastructure could include mechanisms like a centralised engagement registry to facilitate cross‐domain collaboration and guide initiatives that span clinical, research and educational settings. These structures should be developed with patient and family partners representing diverse backgrounds and forms of lived experience.

The application of existing frameworks and models that aim to integrate and foster cooperative engagement efforts across domains within institutions and the healthcare system at large is another important future direction. Models such as the Ecology of Engagement [126], the Ottawa Model of Patient Engagement in Research [131] and the McCain Model of Youth Engagement [24] offer guidance and tangible structures (e.g., dedicated engagement offices, training supports, centralised processes for matching patient partners with investigators, and mentorship pathways) to facilitate effective, cross‐domain engagement. These models emphasise the value of institutional commitment: without centralised leadership, protected funding and consistent processes, engagement remains ad hoc, inequitable and unsustainable. Future research could explore the application, reporting and evaluation of such models within paediatric institutions where engagement spans clinical care, education and research.

## 5 Strengths and Limitations

This review benefited from a multidisciplinary team that included researchers, clinicians and three youth and family partners, some in dual roles, whose contributions strengthened the relevance, interpretation and applicability of the findings. Partner involvement shaped screening decisions, contextualised results and ensured the synthesis reflected priorities important to paediatric patients and families. While the grey literature searching presented itself as a challenge in terms of finding relevant results, we concluded that conducting this search incorporated a desired transparency and strength to our review. Several limitations must also be acknowledged. This scoping review only examined articles published in English due to a lack of resourcing for translation services. Eligibility criteria were refined during screening, as outlined in Appendix B: S2, creating the possibility that some relevant studies were unintentionally excluded. Further, the exclusion of articles at the Inform level of the IAP2 Spectrum could lead to missed insights. The potential value of these articles was discussed after Level 1 screening was complete, and while we tried to remain open to their inclusion during Level 2 screening, we did not identify any meeting our criteria (as they may have been excluded at Level 1). Finally, because all three youth and family partners had existing ties to the institution in which the review was conducted, the perspectives informing this review, while valuable, may not fully represent the broader diversity of partner experiences across other institutions or communities. Nevertheless, this strategy enabled the inclusion of patient and family partners with both strong research interests and valuable lived experience, enhancing the overall quality and depth of the review.

## 6 Future Directions

Given this review's findings regarding often siloed engagement efforts within paediatric institutions, research exploring engagement approaches that span clinical care, education and research is needed. Integrated models and frameworks have the potential to improve efficiency and, in turn, enhance sustainability. Future efforts should embed EDI principles more intentionally and consistently to ensure engagement reflects the needs and experiences of a broader range of patients and families. There is also a pressing need for youth‐led and family‐led initiatives, rather than engagement that positions young people solely as collaborators or advisors. Projects that place youth in leadership roles (e.g., shaping priorities, steering decisions and directing project aims) are uncommon in practice, but essential for meaningful and sustainable engagement. Additionally, improving the quality and consistency of reporting on engagement processes, partner roles and outcomes will further enhance transparency and comparability across studies. At an institutional level, advancing engagement requires dedicated infrastructure, investment and integration across domains, including shared onboarding, compensation pathways, training supports and mechanisms for cross‐departmental coordination.

## 7 Conclusion

This scoping review provides the first comprehensive synthesis of how paediatric patient, family and caregiver engagement is enacted across clinical care, education and research settings. Clear commonalities emerged across domains, including the predominance of small engagement groups, the frequent use of co‐development and feedback roles, and a shared tendency to operate at the *Collaborate* level of the IAP2 Spectrum. At the same time, notable distinctions were identified, including differences in terminology, evaluation practices and reporting of engagement. Together, these findings demonstrate that engagement remains conceptually varied and largely project‐bound across domains. Although many studies aim to involve children, youth and families meaningfully, progress is constrained by the absence of cross‐domain frameworks to guide implementation. Strengthening institutional infrastructure and fostering youth‐ and family‐led approaches will be essential for moving beyond isolated initiatives towards sustainable, system‐level engagement. By prioritising clearer reporting, shared foundations and the inclusion of historically under‐represented groups, paediatric institutions can move towards more coherent, equitable and impactful engagement practices.

## Author Contributions

**Brooke Allemang:** conceptualisation, funding acquisition, writing – original draft, methodology, investigation, formal analysis, resources. **Ida Dehmardan:** writing – original draft, data curation, formal analysis, visualisation, investigation, project administration. **Pranshu Maini:** writing – original draft, investigation, formal analysis, visualisation. **Francine Buchanan:** writing – review and editing, supervision, methodology, investigation, formal analysis. **Ivona Novak:** writing – original draft, investigation. **Vanessa Carbone:** investigation, writing – original draft, data curation. **Dalya Kablawi:** writing – review and editing, investigation, data curation, formal analysis. **Lin Li:** writing – review and editing, data curation, formal analysis, investigation. **Linda Nguyen:** writing – review and editing, investigation, data curation, formal analysis. **Kimberly Courtney:** writing – review and editing, validation. **Jessie Cunningham:** writing – review and editing, validation. **Iveta Lewis:** writing – original draft, validation. **Carla P. Southward:** investigation. **Kristin Cleverley:** writing – review and editing, supervision, validation, formal analysis. **Sarah Munce:** formal analysis, supervision, validation, writing – review and editing. **Alene Toulany:** writing – review and editing, validation, supervision, formal analysis.

## Disclosure

The funding source did not support the study design, collection, data analysis and interpretation, manuscript preparation, or the decision to submit the article for publication.

## Ethics Statement

Ethical approval was not required for this study as it is a scoping review of publicly available literature and did not involve the collection of primary data or identifiable human participant information.

## Conflicts of Interest

The authors declare no conflicts of interest.

## Supporting information


Appendix A

Appendix B

Appendix C

Supporting File 1

Supporting File 2

Supporting File 3

## Data Availability

Data sharing not applicable to this article as no datasets were generated or analysed during the current study. This scoping review is based on data extracted from published literature and publicly available sources. All data generated or analysed during this study are included in this published article and its Supporting Information. Additional data are available upon reasonable request from the corresponding author.
