# Supplementary material for: The Many Faces of Engagement: A Scoping Review of Paediatric Patient and Family Engagement in Clinical Care, Education and Research
Source: Health Expect. 2026 Aug 18;29(4):e70756. doi: 10.1111/hex.70756 (PMC13486972; doi:10.1111/hex.70756)
Supplement: Supplementary file 1 [file HEX-29-e70756-s001.docx]

**A) Study Characteristics**

Setting

| Clinical Care | Any hospital, health centre, clinic, or specialized unit where the primary focus is delivering physical or mental health care (e.g., SickKids, Holland Bloorview, CAMH) |
| --- | --- |
| University | Any university or academic faculty/department, including medical schools or schools of nursing |
| Research Centre | Any independent or university-affiliated research institute, consortium, or collaborative research network whose primary focus is conducting or coordinating research (e.g., CanChild Centre for Childhood Disability Research, OMERACT, Pediatric Emergency Research Canada) |
| Community-based | Any community-facing organization, advocacy group, advisory group, school, youth program, or non-profit service provider where engagement occurs outside of hospitals/universities. This can operate at local, regional, national, or international levels. Primary function is to provide services, advocacy, peer support, or grassroots input. (e.g., mindyourmind, Canadian Arthritis Patient Alliance, Childhood Cancer International) |
| Government | Any national, regional, or state health authority, ministry, or government-funded service that manages or delivers healthcare at the system level (e.g., NHS, Western Australia Child and Adolescent Health Service, Ontario Ministry of Health initiatives) |

Study Methodology

| Qualitative | Collect and analyze non-numerical data (i.e. interviews, focus groups, workshops, written reflections) to explore experiences, perspectives, or processes. Typically uses thematic, content, or framework analysis |
| --- | --- |
| Mixed Methods | Studies that collect and integrate both qualitative and quantitative data within a single project to address related research questions. Data types are analyzed separately and then merged or compared |
| Multi Method | Studies that use two or more distinct methods (e.g., interviews, workshops, surveys) within the same paradigm (purely qualitative in this review) but do not integrate data across methods |
| JLA (James Lind Alliance) | A structured priority-setting partnership process developed by the James Lind Alliance. It combines surveys, evidence checks, and consensus workshops with patients, carers, and clinicians to identify top research priorities |
| Delphi | Consensus-building studies using iterative rounds of surveys or ratings, often completed anonymously by experts or stakeholders, to refine and prioritize items until agreement is reached |
| Reflective | Papers that analyze and describe the process or experience of conducting research or engagement. Typically focuses on lessons learned, challenges, and recommendations |
| RCT (Randomized Control Trial) | Studies that systematically apply improvement frameworks (Plan-Do-Study-Act cycles, CoIIN) to test, implement, and refine healthcare processes or outcomes in real-world settings. Focused on improvement, not generalizable research |
| Quality Improvement | Studies that systematically apply improvement frameworks (Plan-Do-Study-Act cycles, CoIIN) to test, implement, and refine healthcare processes or outcomes in real-world settings. Focused on improvement, not generalizable research |
| Other | Does not neatly fit into any of the preexisting categories |

Research Objective

| Co-Development | To collaboratively create or refine tools, interventions, or resources |
| --- | --- |
| Evaluation and Usability Testing | To assess the acceptability, feasibility, or effectiveness of a product or approach |
| Engagement Process Evaluation | To examine or reflect on the process and impact of engagement itself |
| Experience Exploration | To understand lived experiences, perceptions, or needs of youth, parents, or families |
| Priority Identification | To identify or rank research or service priorities |
| Technology and Digital Health Innovation | To design or improve apps, e-learning, digital tools, or virtual care models |
| Decision-Making Support | To develop frameworks or tools to aid patient, family, caregiver and/or clinician decision processes |
| Capacity Building and Training | To strengthen readiness or skills for patient-oriented research or engagement |
| Transition and Continuity of Care | To improve movement from pediatric to adult or cross-service care |
| Consensus and Outcome Frameworks | To establish shared outcomes, measures, or quality indicators |
| Equity and Inclusion in Service Design | To address underserved or diverse populations in health research or care (beyond condition-specific contexts) |
| Knowledge Translation and Dissemination | To share findings or create tools to inform research, policy, or practice |

**B) Study Population Characteristics**

**Supplemental File 1:** Codebook of Extracted Data

Condition Type

| Mental | Psychiatric or mental health disorders classified in the *DSM-5* (e.g., depression, anxiety disorders, schizophrenia) |
| --- | --- |
| Neurodevelopmental | Neurodevelopmental disorders as defined in the *DSM-5* (e.g., autism spectrum disorder, intellectual disability, ADHD, and learning disorders) |
| Developmental | Developmental disabilities not classified under *Neurodevelopmental Disorders* in the *DSM-5* (e.g., cerebral palsy, Down syndrome, spina bifida, and other congenital syndromes) |
| Physical | Medical or biological conditions such as asthma, diabetes, cancer, epilepsy, and chronic kidney disease |
| Not specified | Refers to cases where the condition type was not specified or too broad to categorize |

**C) PFC Partner Characteristics**

Recruitment Methods

| Existing Networks | Recruitment that draws on pre-established connections, whether through formal advisory bodies, councils, or organizations, or through informal personal, professional, or institutional relationships already known to the research team |
| --- | --- |
| Purposeful/Targeted | Recruitment directed at specific places or groups chosen for relevance, even if the research team didn’t already have a relationship there. Putting posters in clinics, lab websites, schools, or community agencies where the population of interest is likely to be found |
| Open Call | Recruitment through broad, public-facing announcements where anyone meeting criteria can volunteer (social media, websites, newsletters) |
| Convenience | Recruitment that relies on people who are easiest to reach or already accessible to the research team, rather than using systematic or representative methods |
| Snowball | Individuals are identified through referrals from existing participants or contacts. Each recruited individual helps recruit additional participants |

EDI in Recruitment

| Geographic Inclusion | Recruitment strategies aimed at reaching those from specific or various geographic areas (cities/provinces/states, rural/remote/underserved) to ensure representation |
| --- | --- |
| Representation-Focused | Recruitment strategies that explicitly aim to engage a broad, equitable, and inclusive range of individuals |
| Targeted Outreach (Marginalized) | Recruitment through specific organizations, groups or networks (indigenous-led organizations, youth councils, advocacy groups) |
| Literacy Inclusion | Recruitment methods that accommodate different literacy levels or include those with varying literacy levels (oral vs written consent, simplified materials like consent forms) |
| Flexible/Inclusive Criteria | Using broad or self-identification approaches instead of strict definitions (patients defined by self-identification rather than medical records) to avoid excluding people who don’t fit narrow criteria |
| System/Service Access Incusion | Recruitment of individuals who may not access formal healthcare or social service systems (e.g., undocumented individuals, those without health cards and those outside of mainstream structures) |
| Language Inclusion | Ensures individuals who do not speak English are included and/or provides recruitment materials such as consent forms or call-out posters in other languages (e.g., translation services, offering surveys in different languages) |
| Sex and Gender Inclusion | Recruitment strategies that ensure balance or representation across sexes and genders (e.g., purposive sampling for male/female/nonbinary participants) |
| Patient-Oriented Research Experience | Recruitment strategies that intentionally include those with differing levels of prior exposure to research and/or research engagement |
| Age Inclusion | Recruitment strategies that ensure representation across different age groups (e.g., children, youth, adults, older adults) or that avoid exclusion based on age |
| Cultural/Racial/Ethnic Inclusion | Recruitment strategies that intentionally engage individuals from specific cultural, racial, or ethnic groups |
| Condition Variety/Severity | Recruitment strategies that intentionally engage individuals with varying conditions and condition severity |

**D) Engagement Strategies**

Methods of Engagement

| Workshops* | Structured, participatory group activities designed to generate discussion, ideas, or solutions collaboratively. Includes training and educational sessions where individuals engage interactively to learn or co-develop knowledge or skills |
| --- | --- |
| Focus Groups* | Group discussions used to explore individual perspectives, experiences, or needs on a specific topic. Typically involve guided questions and shared dialogue among multiple individuals |
| Interviews* | One-on-one or small group structured or semi-structured discussions to gather detailed feedback or experiences |
| Meetings | Team or project gatherings used to exchange information, discuss progress, or make collaborative decisions among team members, partners, or stakeholders. Includes regular or ad hoc meetings that are not explicitly labeled as workshops, committee meetings, focus groups, etcetera |
| Surveys/Questionnaires* | Structured tools used to collect individual feedback, rank options, or assess perspectives |
| Consensus and Priority-Setting Methods* | Structured processes to achieve group consensus or identify or rank priorities (including JLA and Delphi methodology) |
| Advisory Groups | Ongoing or periodic consultative bodies offering structured input or oversight |
| Steering Committees/Governance Meetings | Groups responsible for providing strategic direction, oversight, or project governance |
| Co-Creation Activities | Collaborative design activities where individuals directly contribute to the creation or improvement of interventions, materials, or tools |
| Testing and Evaluation | Methods that involve individuals testing, piloting, or evaluating tools, processes, or interventions |
| Document Review and Written Feedback | Review, comment on, or edit written materials such as reports, surveys, or drafts |
| Asynchronous Collaboration | Means of engagement that happened asynchronously. Includes email correspondence, shared online workspaces (Google Docs, Slack, Teams) or other asynchronous platforms for reviewing, commenting, or co-creating materials |

* Includes facilitation of method as well as involvement

Frequency of Engagement

| One-time | Engagement occurred only once (within one day, a single workshop, consultation, or interview) |
| --- | --- |
| Few/Limited Sessions | 2 to 5 sessions |
| Multiple Sessions | 6+ sessions, often tied to multiple engagement sessions but still a finite amount |
| Regular – Monthly | Explicitly described engagement as occurring every month |
| Regular – Quarterly | Explicitly described engagement as occurring every ~3 months or ‘quarterly’ |
| Regular – Weekly/Biweekly | Explicitly described engagement as occurring every week or every other week |
| Regular – Other | A clear pattern that doesn’t neatly fit into any of the preexisting categories (“every 6 weeks,” “twice a year”) |
| Ad-hoc | No set schedule; engagement happened when necessary or opportunistically |
| Ongoing | Continuous engagement described, but without a stated cadence or count |

Partner Motivators

| Altruism and Improving Care | Desire to help others, improve services, or enhance care |
| --- | --- |
| Personal Growth and Connection | Seeking personal benefits like learning, skill development, career advancement, or connecting with peers for mutual support |
| Lived Experience and Advocacy | Sharing personal stories or negative experiences to shape research, identify needs, or highlight gaps in care |
| Intrinsic Enthusiasm | Motivation stemming from enthusiasm, energy, or creative drive to codesign, contribute |

EDI in Engagement

| Flexible Scheduling | Avoiding holidays, accommodating scheduling conflicts, offering after-hours sessions or one-on-ones |
| --- | --- |
| Cultural Responsiveness | Adapting food, materials, timing, and language to respect participants’ cultural practices and contexts |
| Remote and Multi-Modal Engagement | Remote and multi-modal options for engagement such as virtual, phone, in-person, or hybrid formats |
| Appropriate Venues | Engagement was held in neutral, safe, or familiar settings |
| Psychological Safety and Safe Spaces | Ensured individuals could share openly without fear of judgment or harm; included practices such as relieving emotionally risky duties or reassigning roles, providing emotional support, and fostering stigma-free environments |
| Accessible Communication and Inclusive Formats | Provided technology support, accessible materials, plain language communication, interpreters, AAC devices, literacy accommodations, flexible formats, and adaptations to age, developmental stage, and communication needs |
| Practical and Logistical Supports | Provided tangible resources such as transportation, parking, meals, and reimbursement to reduce barriers to participation |
| Variations in Group Structuring | Engagement was organized into small or separate groups to ensure youth and parent/caregiver voices were represented and perspectives included |

Training Provided

| Research Training | Training focused on research-related skills and knowledge (research methods, ethics, data collection, analysis, qualitative coding, academic writing) |
| --- | --- |
| Facilitation Training | Training or practice related to facilitating workshops, interviews, focus groups, or peer-support activities (communication skills, managing group dynamics, safeguarding, addressing participant distress) |
| Support/Capacity-Building | Informal or embedded training provided within the engagement process (preparation sessions, technology testing, etcetera) |
| Specialized Training | Training in topic specific knowledge outside of research methods (asthma knowledge, adolescent health, robotics) |
| Project/Process Orientation | Orientation or guidance that explained project goals, expectations, procedures, or roles, often through introductory sessions, ongoing meetings, or one-to-one support rather than structured training |

Recognition Provided

| Remuneration | Direct monetary or monetary-equivalent compensation (cash payments, honoraria, stipends, gift cards, shopping vouchers) |
| --- | --- |
| Co-authorship | Co-authorship on publications, or being named as a Co-Investigator or Co-Applicant |
| Education Credits | Training-related recognition that counts toward academic development (student placement hours, practicum credit, receiving a certificate of completion) |
| Written Acknowledgment | Recognition of contributions through being named or grouped in writing within project materials, reports, publications, newsletters or lab/project websites |

**E) Methods of Contribution**

Roles and Responsibilities

| Topic/Priority Setting | Identifying or ranking research questions, gaps, or priorities to achieve consensus |
| --- | --- |
| Recruitment and Outreach | Supporting participant recruitment (e.g., sharing materials in networks, advising on compensation/honoraria, co-creating recruitment materials) |
| Data Analysis and Interpretation | Contributing to data interpretations and codebook development (e.g., reviewing transcripts, coding, selecting quotes) |
| Data Collection Participation | Partaking in focus groups, interviews, workshops where lived experiences and personal stories are being shared (as an interviewee) |
| Study Conceptualization and Protocol Development | Refining research questions, contributing to ethics applications, protocols and general scope (e.g., Terms of Reference documents, REBs) |
| Knowledge Translation and Dissemination | Co-authoring manuscripts, blogs, infographics, etcetera. Presenting at conferences, community events, or webinars. |
| Co-Develop Tools/Materials | Developing items related to the research study, such as surveys, interview guides, resources, decision aids |
| Feedback and Review | Reviewing/approving drafts, prototypes, language, design, and usability. Providing iterative feedback across development stages |
| Sharing Lived Experiences | Sharing lived/living experience expertise (patient/caregiver/family/youth perspective) |
| Governance and Decision Making | Serving on steering or advisory committees, contributing to strategic planning and decision-making, and taking on leadership roles to guide project direction |
| Intervention Developmental and Refinement | Individuals shape or improve the programs, services, or supports that are being tested or implemented as the intervention itself. These outputs are the intervention under study (content, delivery, format), not research facilitation tools |
| Co-Facilitation | Co-leading modules, focus groups, or workshops. Facilitating discussions and guiding younger individuals |

**F) Impacts of Engagement**

Impacts on Research

| Increased Relevance to End Users | Research findings, outputs, or interventions became higher quality and more meaningful by incorporating context, credibility, and validity, and in some cases by integrating diverse perspectives to better reflect the needs of specific end-user groups |
| --- | --- |
| Outputs Grounded in Lived Experiences | Outputs, findings, interventions are directly and explicitly shaped by lived experiences/perspectives of partners |
| Increased Methodological Rigor | Engagement strengthened the research process and/or credibility, validity, and robustness of research by enhancing data quality, improving interpretation, and/or ensuring findings were more comprehensive and trustworthy |
| Broadened Accessibility of Research Outputs | Materials and interventions were modified to be more understandable and inclusive, to ensure comprehensive engagement |
| Impacted Recruitment and Reach | Engagement enhanced recruitment and retention and/or broadening the reach of research findings by ensuring outputs were disseminated in ways that resonated with the target populations |

Impacts on Partners

| Felt Comfortable to Express Perspectives | Partners felt encouraged to share their views and experiences, as well as safe and heard |
| --- | --- |
| Felt Contributions Meaningfully Impacted Research | Partners felt their input changed the research process, protocol or intervention |
| Developed Appreciation of Authentic Engagement | Reported enjoyment and/or appreciation of the engagement process |
| Capacity Development | Partners gained knowledge, built skills (e.g., in data collection, analysis, interviewing), and experienced professional development (e.g., recognition, conference exposure, training, certifications) |
| Felt Empowered | Developed interest in research and future partnership opportunities; increased confidence to engage in other partnerships |
| Felt Valued | Reported feeling like an equal team member and that feedback was meaningfully considered and incorporated |
| Identified/Strengthened Connection within Community | Felt emotionally and/or psychologically supported through engagement; developed connections with peers sharing similar experiences, reducing feelings of isolation. |

Impacts on Researchers

| Built Engagement Capacity/Proficiency | Gained experience in reaching consensus, learned engagement best practices, enhanced accountability to meaningful patient partnership, and broadened access to communities |
| --- | --- |
| Understood Partner’s Perspectives | Gained deeper understanding of partners’ experiences; broadened understanding of the needs and challenges of specific population groups |
| Inspired Personal Reflection of Values and Research Process | Recognized the value of building partnerships; broadened perspectives on how patient partners experiences inform research and incorporated changes to research protocols because of partnerships |
