# Supplementary material for: The Many Faces of Engagement: A Scoping Review of Paediatric Patient and Family Engagement in Clinical Care, Education and Research
Source: Health Expect. 2026 Aug 18;29(4):e70756. doi: 10.1111/hex.70756 (PMC13486972; doi:10.1111/hex.70756)
Supplement: Supplementary file 2 [file HEX-29-e70756-s002.docx]

# **Supplemental File 3. Grey Literature Search Strategy**

# Strategy 1. Targeted Website Browsing/Searching (ie search 1 website at a time)

Browse and/or search (using a variety of identified words) the websites of organizations (government, health organizations, NGOs, universities, research centres, etc.) that publish documents relevant to the research question.

## i) Website Browsing Documentation:

| **Date** | **Organization name**  *Ex. Public Health Ontario* | **URL**  *Ex. https://www.publichealthontario.ca/en/Pages/default.aspx* | **# of items screened (uploaded to citation management software)**  *Ex. 3* |
| --- | --- | --- | --- |
| July 31, 2025 | **CADTH** | https://www.cda-amc.ca/search?s=patient%20engagement%20pediatrics | 0 |
|  |  | https://www.cda-amc.ca/search?s=patient-centred%20research%20%20pediatrics&op=OR |  |
|  |  | https://www.cda-amc.ca/search?s=health-care%20delivery%20%20pediatrics&op=OR |  |
|  | **RAND** | [Search rand.org \| RAND](https://www.rand.org/search.html?q=patient+engagement+pediatrics) |  |
|  |  | [Search rand.org \| RAND](https://www.rand.org/search.html?q=patient-oriented+research+pediatrics&sortby=relevance&rows=12) |  |
|  |  | [Search rand.org \| RAND](https://www.rand.org/search.html?q=health-care+delivery+pediatrics&sortby=relevance&rows=12) |  |
|  | **Cerebral Palsy Research Network** | [You searched for health care delivery pediatrics \| Cerebral Palsy Research Network](https://cprn.org/?s=health+care+delivery+pediatrics) |  |
|  |  | [You searched for patient engagement pediatrics \| Cerebral Palsy Research Network](https://cprn.org/?s=patient+engagement+pediatrics) |  |
|  |  | [You searched for patient research pediatrics \| Cerebral Palsy Research Network](https://cprn.org/?s=patient+research+pediatrics) |  |
|  | Overton – will explain / paywall |  |  |
|  | **CIHI** | https://www.cihi.ca/en/search?query=health+care+delivery+pediatrics |  |
|  |  | https://www.cihi.ca/en/search?query=PATIENT+ENGAGEMENT+pediatrics |  |
|  |  | https://www.cihi.ca/en/search?query=patient-oriented+research+pediatrics |  |
|  | **Health Canada** | https://www.canada.ca/en/sr/srb.html?q=patient+engagement+pediatrics&wb-srch-sub= |  |
|  |  | https://www.canada.ca/en/sr/srb.html#q=patient-oriented%20research%20pediatrics |  |
|  |  | https://www.canada.ca/en/sr/srb.html#q=patient-oriented%20research%20health%20care%20delivery%20pediatrics |  |
|  | **Health Quality Ontario duckduckgo** | See below the two links they wouldn’t fit here |  |
|  | **Oister** | See below the two links they wouldn’t fit here |  |
|  | **OpenDoar** | No results |  |
|  | **Proquest – must have utorid** | <https://www.proquest.com/results/F19DCE4B3AB144D5PQ/1?accountid=14771>  ((healthcare delivery) AND (community-based participatory research) AND (patient centred care research) |  |
|  | **Google Scholar** | https://scholar.google.ca/scholar?q=delivery+of+healthcare+community-based+research+patient-centred+research&hl=en&as_sdt=0&as_vis=1&oi=scholart |  |

**Health Quality Ontario**

**https://www.hqontario.ca/SearchResults/Search-Results#?cludoquery=patient%20-oriented%20research%20health%20care%20delivery%20pediatrics&cludopage=1&cludorefurl=https%3A%2F%2Fwww.hqontario.ca%2FEvidence-to-Improve-Care%2FHealth-Technology-Assessment&cludorefpt=Health%20Technology%20Assessment%20-%20Health%20Quality%20Ontario%20(HQO)&cludoinputtype=standard**

[**https://www.hqontario.ca/SearchResults/Search-Results#?cludoquery=patient%20engagement%20health%20care%20delivery%20pediatrics&cludopage=1&cludorefurl=https%3A%2F%2Fwww.hqontario.ca%2FEvidence-to-Improve-Care%2FHealth-Technology-Assessment&cludorefpt=Health%20Technology%20Assessment%20-%20Health%20Quality%20Ontario%20(HQO)&cludoinputtype=standard**](https://www.hqontario.ca/SearchResults/Search-Results#?cludoquery=patient%20engagement%20health%20care%20delivery%20pediatrics&cludopage=1&cludorefurl=https%3A%2F%2Fwww.hqontario.ca%2FEvidence-to-Improve-Care%2FHealth-Technology-Assessment&cludorefpt=Health%20Technology%20Assessment%20-%20Health%20Quality%20Ontario%20(HQO)&cludoinputtype=standard)

**Duckduckgo**

**https://duckduckgo.com/?t=h_&q=delivery+of+health+care+AND+patient+centred+research+AND++community+based+participatory+research+AND+adolescents&ia=web**

**Oister**

**https://oaister.on.worldcat.org/search?queryString=healthcare%20delivery%20AND%20community%20based%20participatory%20research%20&bookReviews=off&clusterResults=true&groupVariantRecords=false**

**https://oaister.on.worldcat.org/search?queryString=healthcare%20delivery%20AND%20patient-centred%20research&bookReviews=off&clusterResults=true&groupVariantRecords=false**
