# Supplementary material for: The Many Faces of Engagement: A Scoping Review of Paediatric Patient and Family Engagement in Clinical Care, Education and Research
Source: Health Expect. 2026 Aug 18;29(4):e70756. doi: 10.1111/hex.70756 (PMC13486972; doi:10.1111/hex.70756)
Supplement: Supplementary file 3 [file HEX-29-e70756-s003.docx]

**Supplemental File 2:** Overview of Included Studies

| **Study** | **Country** | **Domain** | **Setting** | **Condition Type** | **Partner Type and Total #** | **IAP2 Category** |
| --- | --- | --- | --- | --- | --- | --- |
| Akama (2023) | Kenya | Clinical | Research Centre | Physical | Patient (3) | Collaborate |
| Ali (2024) | Canada | Clinical | Clinical Care | Mental | Patient/Caregiver (10) | Collaborate |
| Allemang (2023) | Canada | Research | Clinical Care / University | Mental/Neurodevelopmental/Physical | Patient (5) | Collaborate |
| Allemang (2023) | Canada | Research | Clinical Care | Mental/Physical | Patient (5) | Collaborate |
| Amirav (2017) | Canada | Research | Clinical Care / University | Physical | Patient / Caregiver (4) | Involve |
| Aufegger (2020) | United Kingdom | Clinical | University | Not specified | Patient / Caregiver (47) | Consult / Involve |
| Bailey (2024) | Canada | Research | Clinical Care / Research Centre / University | Not specified | Patient (11) | Collaborate / Empower |
| Balay-Dustrude (2023) | America / Canada | Research | Research Centre | Physical | Patient / Caregiver (37) | Involve |
| Bialy (2018) | Canada | Research | Research Centre | Not specified | Caregiver (22) | Involve |
| Bird (2021) | Canada | Research | Clinical Care | Physical | Caregiver (2) | Collaborate |
| Birnie (2019) | Canada | Research | Clinical Care | Physical | Patient / Caregiver (5) | Collaborate |
| Bluebond-Langner (2024) | United Kingdom | Clinical | Clinical Care / University | Physical | Caregiver (21) | Consult / Involve |
| Boote (2016) | United Kingdom | Clinical | Clinical Care | Physical | Caregiver (2) | Consult** |
| Brewer (2019) | America | Research | Research Centre | Not specified | Caregiver (19) | Involve |
| Butler (2019) | United Kingdom | Clinical | Research Centre | Physical | Patient / Caregiver (Not specified) | Consult |
| Cadwgan (2024) | United Kingdom | Research | Community-based / Government | Physical | Patient / Caregiver (8) | Involve |
| Campbell (2020) | Canada | Research | Clinical Care / Community-based | Mental | Patient / Caregiver (~34) | Consult |
| Campbell (2022) | Canada | Research | University | Physical | Patient / Family (Not specified) | Consult / Involve |
| Carrera Diaz (2024) | America | Research | Clinical Care | Physical | Patient / Family (Not specified) | Involve |
| Carter (2012) | United Kingdom | Research | Community-based / Government | Physical | Patient / Caregiver (Not specified) | Involve |
| Cavens (2022) | Australia | Research | University | Developmental | Patient (1) | Collaborate |
| Chan (2020) | America | Clinical | Clinical Care | Physical | Caregiver (10) | Involve |
| Cheng (2021) | Australia | Clinical | Research Centre | Mental | Patient / Family / Caregiver (83) | Involve |
| Cleverley (2022) | Canada | Research | Clinical Care | Mental | Patient / Family / Caregiver (6) | Involve / Collaborate |
| Costello & Dorris (2020) | Ireland | Research | Research Centre / University | Physical | Patient / Family (15) | Involve |
| Cousino (2023) | America | Clinical | Clinical Care | Physical | Caregiver (Not specified) | Involve |
| Crosby (2017) | America | Clinical | University | Physical | Patient (70) | Involve |
| Cross (2022) | Canada | Research | University | Developmental | Patient / Caregiver (Not specified) | Involve |
| DeCourcey (2021) | America | Clinical | Clinical Care | Physical | Patient / Caregiver (8) | Consult |
| Dewa (2021) | United Kingdom | Research | Research Centre | Mental | Patient (5) | Collaborate |
| Dong (2023) | Canada | Research | Clinical Care | Developmental / Neurodevelopmental | Patient / Caregiver (5) | Collaborate / Empower |
| Dosso (2024) | Canada | Clinical | Clinical Care / University | Mental | Patient / Caregiver (8) | Involve |
| Dovey-Pearce (2019) | United Kingdom | Research | Clinical Care | Developmental / Physical | Patient (Not specified) | Collaborate / Empower |
| Drury (2022) | United Kingdom / Ireland | Research | University | Physical | Patient / Family / Caregiver (387) | Involve |
| Eisler (2016) | United Kingdom | Clinical | Clinical Care | Physical | Patient / Family / Caregiver (Not specified) | Involve / Collaborate |
| Fawole (2021) | America / Canada | Clinical | Research Centre | Physical | Patient / Caregiver (6) | Involve / Collaborate |
| Fink (2020) | America | Clinical | University | Physical | Patient / Family / Caregiver (Not specified) | Involve |
| Finlay-Jones (2023) | Australia | Research | Government | Developmental / Neurodevelopmental / Physical | Patient / Caregiver (18) | Involve |
| Gerchow (2024) | America | Clinical | Community-based | Physical | Patient (10) | Involve |
| Gibson (2021) | America | Research | Clinical Care | Physical | Patient / Family / Caregiver (Not specified) | Involve |
| Gonzalez (2021) | Canada | Research | Clinical Care | Developmental / Neurodevelopmental / Physical | Caregiver (1) | Collaborate |
| Gonzalez (2023) | Canada | Research | Clinical Care | Developmental | Caregiver (2) | Collaborate |
| Griesemer (2020) | America | Clinical | University | Physical | Caregiver (7) | Involve / Collaborate |
| Guo (2024) | Australia | Research | Research Centre / University | Mental | Patient (8) | Collaborate |
| Gustavsson (2015) | Sweden | Clinical | Clinical Care | Physical | Caregiver (12) | Involve |
| Hackett (2018) | Canada | Clinical | University | Mental | Patient / Caregiver (11) | Involve |
| Handra (2022) | Canada | Clinical | Clinical Care / University | Developmental | Caregiver (3) | Collaborate |
| Hannon (2018) | America | Clinical | Research Centre / University | Physical | Patient / Caregiver (12) | Involve |
| Holtz (2017) | America | Clinical | University | Physical | Patient / Caregiver (12) | Consult |
| Houlihan (2024) | America | Clinical | University | Physical | Caregiver (Not specified) | Involve |
| Huggett (2017) | Canada | Clinical | Clinical Care / Community-based / Government | Mental | Patient (9) | Collaborate |
| Keir (2023) | Canada | Research | Clinical Care | Physical | Patient (35) | Consult / Involve |
| Kim (2023) | Canada | Research | University | Physical | Caregiver (2) | Collaborate / Empower |
| Kim (2024) | Canada | Research | Clinical Care / University | Physical | Caregiver (1) | Collaborate / Empower |
| Kirk (2023) | United Kingdom | Research | University | Mental | Patient (6) | Collaborate |
| Knochel (2022) | Germany / Switzerland | Clinical | Clinical Care | Physical | Caregiver (9) | Involve |
| Kramer & Schwartz (2018) | America | Clinical | University | Developmental / Neurodevelopmental | Patient (8) | Collaborate |
| Lakhanpaul (2022) | United Kingdom | Clinical | Community-based | Physical | Caregiver (2) | Collaborate |
| Leake (2024) | Australia | Education | University | Physical | Patient (7) | Collaborate* |
| Leblond (2024) | United Kingdom | Research | Government | Not specified | Caregiver (105) | Involve |
| Liu (2020) | America | Clinical | Clinical Care | Physical | Caregiver (2) | Collaborate |
| Livanou (2024) | United Kingdom | Clinical | Clinical Care | Mental / Neurodevelopmental | Patient / Caregiver (15) | Involve |
| Lopez-Vargas (2018) | Australia | Research | Community-based / University | Physical | Patient / Caregiver (22) | Involve |
| Ludlow (2023) | Australia | Clinical | University | Mental | Patient (68) | Consult |
| Macarthur (2021) | Canada | Education | Clinical Care | Not specified | Caregiver (Not specified) | Collaborate |
| Mackworth-Young (2022) | Zimbabwe | Clinical | Community-based | Physical | Patient (12) | Consult / Involve |
| Manning (2017) | United Kingdom | Research | University | Physical | Patient / Family / Caregiver (11) | Consult / Involve |
| Martin (2023) | Canada | Research | Clinical Care / University | Physical | Patient / Caregiver (18) | Involve |
| Massey (2024) | United Kingdom | Clinical | Clinical Care | Developmental | Caregiver (20) | Collaborate |
| McKenna (2024) | Australia | Clinical | Clinical Care | Mental | Patient (2) | Collaborate |
| McPherson (2016) | Canada | Research | Research Centre | Not specified | Patient / Caregiver (4) | Involve |
| Meagher (2024) | Australia | Clinical | Clinical Care / University | Neurodevelopmental / Physical | Caregiver (13) | Involve |
| Micsinszki (2022) | Canada | Research | Clinical Care / Research Centre | Developmental / Neurodevelopmental / Physical | Patient / Caregiver (Not specified) | Involve / Collaborate |
| Morris (2015) | United Kingdom | Research | Clinical Care / Community-based | Developmental / Neurodevelopmental / Physical | Patient / Caregiver (3) | Involve |
| Narula (2024) | United Kingdom | Research | Clinical Care / University | Physical | Patient / Caregiver (4) | Involve |
| Nguyen (2024) | Canada | Clinical | University | Physical | Family (6) | Collaborate |
| Ostojic (2024) | Australia | Clinical | Clinical Care | Developmental | Patient / Caregiver (Not specified) | Collaborate |
| Oulton (2020) | United Kingdom | Clinical | Clinical Care | Physical | Patient / Family / Caregiver (101) | Involve / Collaborate |
| Pagel (2017) | United Kingdom | Research | Clinical Care | Physical | Family (3) | Involve |
| Pearson (2024) | United Kingdom | Research | Clinical Care / University | Physical | Caregiver (5) | Collaborate |
| Pembroke (2021) | Ireland | Clinical | Clinical Care | Physical | Patient / Caregiver (10) | Collaborate |
| Pembroke (2022) | Ireland | Clinical | Clinical Care | Physical | Patient / Caregiver (10) | Collaborate |
| Phillips (2019) | United Kingdom | Clinical | Clinical Care / University | Physical | Patient / Caregiver (6) | Involve |
| Poku & Pilnick (2022) | Ghana | Research | Clinical Care / Community-based | Physical | Caregiver (20) | Consult |
| Potter (2024) | United Kingdom | Clinical | Clinical Care | Physical | Patient / Caregiver (45) | Involve / Collaborate |
| Radford (2020) | Australia | Clinical | Clinical Care | Physical | Family (26) | Collaborate |
| Randolph (2024) | America | Clinical | University | Physical | Family (Not specified) | Collaborate |
| Samuel (2017) | Canada | Research | University | Physical | Patient / Family / Caregiver (12) | Involve |
| Schwartz & Kramer (2020) | America | Research | University | Developmental / Neurodevelopmental | Patient (8) | Collaborate |
| Seid (2018) | America | Research | Research Centre | Physical | Patient / Caregiver (16) | Collaborate |
| Seko (2024) | Canada | Clinical | Clinical Care | Developmental / Physical | Patient / Family (Not specified) | Collaborate |
| Sheikhan (2023) | Canada | Clinical | Clinical Care | Mental | Patient (5) | Collaborate |
| Sligo (2019) | New Zealand | Research | Community-based | Physical | Patient (8) | Collaborate |
| Sood (2022) | America | Clinical | Clinical Care | Physical | Caregiver (4) | Collaborate |
| Taylor (2022) | United Kingdom | Clinical | Clinical Care / University | Mental / Physical | Caregiver (Not specified) | Collaborate |
| Tsang (2020) | America / Canada | Research | Clinical Care / Community-based | Not specified | Patient / Family (14) | Collaborate |
| Turuba (2022) | Canada | Research | Clinical Care / Community-based | Mental | Patient (14) | Collaborate |
| van Schelven (2021) | Netherlands | Research | Research Centre / University | Not specified | Patient (1) | Consult / Involve |
| van Schelven (2024) | Netherlands | Clinical | Research Centre | Developmental / Physical | Patient (Not specified) | Involve / Collaborate |
| Vella-Baldacchino (2019) | United Kingdom | Research | Clinical Care / University | Physical | Patient / Caregiver (Not specified) | Collaborate |
| Verwoerd (2021) | Netherlands | Research | Clinical Care | Physical | Patient / Caregiver (7) | Collaborate |
| Vickers (2021) | United Kingdom | Clinical | Community-based | Physical | Family (Not specified) | Collaborate |
| Virk (2019) | Canada | Clinical | University | Mental | Patient / Caregiver (38) | Involve |
| Walker (2020) | United Kingdom | Research | Clinical Care | Physical | Patient / Caregiver (18) | Collaborate |
| Walsh (2022) | Canada | Education | Clinical Care | Not specified | Caregiver (4) | Collaborate* |
| Wambua (2019) | Kenya | Clinical | Clinical Care | Physical | Patient (9) | Consult / Involve / Collaborate / Empower* |
| Webbe (2020) | United Kingdom | Research | Clinical Care | Physical | Patient / Caregiver (244) | Involve |
| Weiler-Wichtl (2023) | Austria | Education | Clinical Care / Community-based | Physical | Patient (1) | Collaborate |
| Weiler-Wichtl (2024) | Austria | Research | University | Physical | Patient (4) | Collaborate |
| Wintels (2018) | Netherlands | Research | Clinical Care / Community-based | Developmental | Patient (12) | Collaborate |
| Wong (2023) | America | Research | University | Not specified | Family (Not specified) | Involve |
| Wood (2023) | Canada | Clinical | Clinical Care | Physical | Caregiver (9) | Involve |
| Xafis (2015) | Australia | Clinical | Clinical Care | Physical | Caregiver (6) | Consult |

*IAP2 Spectrum of Public Participation explicitly stated

**Hart’s Ladder of Participation explicitly stated

References

1. Akama EO, Beres LK, Kulzer JL, et al. A youth-centred approach to improving engagement in HIV services: human-centred design methods and outcomes in a research trial in Kisumu County, Kenya. *BMJ Glob Health*. Nov 29 2023;8(11)doi:10.1136/bmjgh-2023-012606

2. Ali AZ, Wright B, Curran JA, Fawcett-Arsenault J, Newton AS. Co-designing discharge communication interventions for mental health visits to the pediatric emergency department: a mixed-methods study. *Res Involv Engagem*. Jun 21 2024;10(1):64. doi:10.1186/s40900-024-00594-y

3. Allemang B, Samuel S, Greer K, et al. Transition readiness of youth with co-occurring chronic health and mental health conditions: A mixed methods study. *Health Expect*. Dec 2023;26(6):2228–2244. doi:10.1111/hex.13821

4. Allemang B, Samuel S, Pintson K, et al. "They go hand in hand": a patient-oriented, qualitative descriptive study on the interconnectedness between chronic health and mental health conditions in transition-age youth. *BMC Health Serv Res*. Jan 2 2023;23(1):2. doi:10.1186/s12913-022-09002-1

5. Amirav I, Vandall-Walker V, Rasiah J, Saunders L. Patient and Researcher Engagement in Health Research: A Parent's Perspective. *Pediatrics*. Sep 2017;140(3)doi:10.1542/peds.2016-4127

6. Aufegger L, Bùi KH, Bicknell C, Darzi A. Designing a paediatric hospital information tool with children, parents, and healthcare staff: a UX study. *BMC Pediatr*. Oct 8 2020;20(1):469. doi:10.1186/s12887-020-02361-w

7. Bailey K, Allemang B, Vandermorris A, et al. Benefits, barriers and recommendations for youth engagement in health research: combining evidence-based and youth perspectives. *Res Involv Engagem*. Sep 2 2024;10(1):92. doi:10.1186/s40900-024-00607-w

8. Balay-Dustrude E, Christensen R, Consolaro A, et al. Defining patient perception of overall well-being and disease activity in the OMERACT Juvenile Idiopathic Arthritis (JIA) core domain set: A report from the JIA working group. *Semin Arthritis Rheum*. Feb 2024;64:152340. doi:10.1016/j.semarthrit.2023.152340

9. Bialy L, Plint AC, Freedman SB, Johnson DW, Curran JA, Stang AS. Pediatric Emergency Research Canada (PERC): Patient/Family-informed Research Priorities for Pediatric Emergency Medicine. *Acad Emerg Med*. Dec 2018;25(12):1365–1374. doi:10.1111/acem.13493

10. Bird M, McGillion M, Chambers EM, et al. A generative co-design framework for healthcare innovation: development and application of an end-user engagement framework. *Res Involv Engagem*. Mar 1 2021;7(1):12. doi:10.1186/s40900-021-00252-7

11. Birnie KA, Dib K, Ouellette C, et al. Partnering For Pain: a Priority Setting Partnership to identify patient-oriented research priorities for pediatric chronic pain in Canada. *CMAJ Open*. Oct–Dec 2019;7(4):E654–e664. doi:10.9778/cmajo.20190060

12. Bluebond-Langner M, Wakeling S, Vincent K, Butler A, Brown K, Jowett V. British Congenital Cardiac Association Fetal Cardiology Standards: impact of Patient and Public involvement exercise on the development of clinical standards. *Cardiol Young*. Jun 2024;34(6):1206–1210. doi:10.1017/s1047951123004298

13. Boote J, Julious S, Horspool M, Elphick H, Smithson WH, Norman P. PPI in the PLEASANT trial: involving children with asthma and their parents in designing an intervention for a randomised controlled trial based within primary care. *Prim Health Care Res Dev*. Nov 2016;17(6):536–548. doi:10.1017/s1463423616000025

14. Brewer SE, Crump NM, O'Leary ST. Patient-Centered Research Priorities: A Mixed-Methods Approach from the Colorado Children's Outcomes Network (COCONet). *J Am Board Fam Med*. Sep–Oct 2019;32(5):674–684. doi:10.3122/jabfm.2019.05.190028

15. Butler DP, De la Torre A, Borschel GH, et al. An International Collaborative Standardizing Patient-Centered Outcome Measures in Pediatric Facial Palsy. *JAMA Facial Plast Surg*. Sep 1 2019;21(5):351–358. doi:10.1001/jamafacial.2019.0224

16. Cadwgan J, Goodwin J, Babcock B, et al. UK research priority setting for childhood neurological conditions. *Dev Med Child Neurol*. Dec 2024;66(12):1590–1599. doi:10.1111/dmcn.16021

17. Campbell A, Hartling L, Plourde V, Scott SD. Parental Knowledge, Self-confidence, and Usability Evaluation of a Web-Based Infographic for Pediatric Concussion: Multimethod Study. *JMIR Pediatr Parent*. May 10 2022;5(2):e36317. doi:10.2196/36317

18. Campbell LA, Lovas D, Withers E, Peacock K. Opening the door: inviting youth and parent perspectives on youth mental health emergency department use. *Res Involv Engagem*. 2020;6:26. doi:10.1186/s40900-020-00204-7

19. Carrera Diaz K, Yau J, Iverson E, et al. Human-centered design approach to building a transition readiness mHealth intervention for early adolescents. *J Pediatr Psychol*. Jan 1 2025;50(1):106–114. doi:10.1093/jpepsy/jsae066

20. Carter B, Coad J, Bray L, et al. Home-based care for special healthcare needs: community children's nursing services. *Nurs Res*. Jul–Aug 2012;61(4):260–8. doi:10.1097/NNR.0b013e31825b6848

21. Cavens C, Imms C, Drake G, Garrity N, Wallen M. Perspectives of children and adolescents with cerebral palsy about involvement as research partners: a qualitative study. *Disabil Rehabil*. Aug 2022;44(16):4293–4302. doi:10.1080/09638288.2021.1900927

22. Chan KH, Misseri R, Carroll A, et al. User-centered development of a hypospadias decision aid prototype. *J Pediatr Urol*. Oct 2020;16(5):684.e1–684.e9. doi:10.1016/j.jpurol.2020.07.047

23. Cheng VWS, Piper SE, Ottavio A, Davenport TA, Hickie IB. Recommendations for Designing Health Information Technologies for Mental Health Drawn From Self-Determination Theory and Co-design With Culturally Diverse Populations: Template Analysis. *J Med Internet Res*. Feb 10 2021;23(2):e23502. doi:10.2196/23502

24. Cleverley K, McCann E, O'Brien D, et al. Prioritizing core components of successful transitions from child to adult mental health care: a national Delphi survey with youth, caregivers, and health professionals. *Eur Child Adolesc Psychiatry*. Nov 2022;31(11):1739–1752. doi:10.1007/s00787-021-01806-6

25. Costello W, Dorris E. Laying the groundwork: Building relationships for public and patient involvement in pre-clinical paediatric research. *Health Expect*. Feb 2020;23(1):96–105. doi:10.1111/hex.12972

26. Cousino MK, May LJ, Smyth L, et al. Patient and parent-reported outcomes in paediatric ventricular assist device support: a multi-center ACTION learning network feasibility and pilot experience. *Cardiol Young*. Nov 2023;33(11):2258–2266. doi:10.1017/s1047951122004048

27. Crosby LE, Ware RE, Goldstein A, et al. Development and evaluation of iManage: A self-management app co-designed by adolescents with sickle cell disease. *Pediatr Blood Cancer*. Jan 2017;64(1):139–145. doi:10.1002/pbc.26177

28. Cross A, Soper AK, Tomas V, et al. Exploring 10 years of dissemination of the F-words for Child Development: A multifaceted case study. *Child Care Health Dev*. Sep 2022;48(5):751–762. doi:10.1111/cch.12983

29. DeCourcey DD, Partin L, Revette A, Bernacki R, Wolfe J. Development of a Stakeholder Driven Serious Illness Communication Program for Advance Care Planning in Children, Adolescents, and Young Adults with Serious Illness. *J Pediatr*. Feb 2021;229:247–258.e8. doi:10.1016/j.jpeds.2020.09.030

30. Dewa LH, Lawrence-Jones A, Crandell C, et al. Reflections, impact and recommendations of a co-produced qualitative study with young people who have experience of mental health difficulties. *Health Expect*. May 2021;24 Suppl 1(Suppl 1):134–146. doi:10.1111/hex.13088

31. Dong SY, Nguyen L, Cross A, et al. Youth engagement in research: exploring training needs of youth with neurodevelopmental disabilities. *Res Involv Engagem*. Jul 10 2023;9(1):50. doi:10.1186/s40900-023-00452-3

32. Dosso JA, Kailley JN, Robillard JM. The League: A person-centred approach to the development of social robotics for paediatric anxiety. *Health Expect*. Feb 2024;27(1):e13981. doi:10.1111/hex.13981

33. Dovey-Pearce G, Walker S, Fairgrieve S, Parker M, Rapley T. The burden of proof: The process of involving young people in research. *Health Expect*. Jun 2019;22(3):465–474. doi:10.1111/hex.12870

34. Drury NE, Herd CP, Biglino G, et al. Research priorities in children and adults with congenital heart disease: a James Lind Alliance Priority Setting Partnership. *Open Heart*. Nov 2022;9(2)doi:10.1136/openhrt-2022-002147

35. Eisler I, Ellison M, Flinter F, et al. Developing an intervention to facilitate family communication about inherited genetic conditions, and training genetic counsellors in its delivery. *Eur J Hum Genet*. Jun 2016;24(6):794–802. doi:10.1038/ejhg.2015.215

36. Fawole OA, Reed MV, Harris JG, et al. Engaging patients and parents to improve mental health intervention for youth with rheumatological disease. *Pediatr Rheumatol Online J*. Feb 23 2021;19(1):19. doi:10.1186/s12969-021-00503-7

37. Fink EL, Maddux AB, Pinto N, et al. A Core Outcome Set for Pediatric Critical Care. *Crit Care Med*. Dec 2020;48(12):1819–1828. doi:10.1097/ccm.0000000000004660

38. Finlay-Jones A, Sampson R, Parkinson A, et al. Priority setting for children and young people with chronic conditions and disabilities. *Health Expect*. Aug 2023;26(4):1562–1574. doi:10.1111/hex.13761

39. Gerchow L, Lanier Y, Fayard AL, Squires A. Cocreating First Steps, a Toolkit to Improve Adolescent Sexual and Reproductive Health Services: Qualitative Human-Centered Design Study With Hispanic and Black Adolescent Mothers in New York City. *JMIR Pediatr Parent*. Nov 19 2024;7:e60692. doi:10.2196/60692

40. Gibson F, Fern LA, Phillips B, et al. Reporting the whole story: Analysis of the 'out-of-scope' questions from the James Lind Alliance Teenage and Young Adult Cancer Priority Setting Partnership Survey. *Health Expect*. Oct 2021;24(5):1593–1606. doi:10.1111/hex.13276

41. Gonzalez M, Ogourtsova T, Zerbo A, et al. Patient engagement in a national research network: barriers, facilitators, and impacts. *Res Involv Engagem*. Mar 8 2023;9(1):7. doi:10.1186/s40900-023-00418-5

42. Gonzalez M, Saxena S, Chowdhury F, et al. Informing the development of the Canadian Neurodiversity Platform: What is important to parents of children with neurodevelopmental disabilities? *Child Care Health Dev*. Jan 2022;48(1):88–98. doi:10.1111/cch.12906

43. Griesemer I, Staley BS, Lightfoot AF, et al. Engaging community stakeholders in research on best practices for clinical genomic sequencing. *Per Med*. Nov 2020;17(6):435–444. doi:10.2217/pme-2020-0074

44. Guo K, Meas D, Mautner D, et al. Guidelines for the design and implementation of youth participation initiatives to safeguard mental health and wellbeing. *Med J Aust*. Nov 18 2024;221 Suppl 10:S34–s40. doi:10.5694/mja2.52485

45. Gustavsson S, Gremyr I, Kenne Sarenmalm E. Designing quality of care--contributions from parents: Parents' experiences of care processes in paediatric care and their contribution to improvements of the care process in collaboration with healthcare professionals. *J Clin Nurs*. Mar 2016;25(5-6):742–51. doi:10.1111/jocn.13050

46. Hackett CL, Mulvale G, Miatello A. Co-designing for quality: Creating a user-driven tool to improve quality in youth mental health services. *Health Expect*. Dec 2018;21(6):1013–1023. doi:10.1111/hex.12694

47. Handra J, Guimond C, Jordan I, et al. A personalized genomic results e-booklet, co-designed and pilot-tested by families. *PEC Innov*. Dec 2022;1:100039. doi:10.1016/j.pecinn.2022.100039

48. Hannon TS, Moore CM, Cheng ER, et al. Codesigned Shared Decision-Making Diabetes Management Plan Tool for Adolescents With Type 1 Diabetes Mellitus and Their Parents: Prototype Development and Pilot Test. *J Particip Med*. May 10 2018;10(2):e8. doi:10.2196/jopm.9652

49. Holtz BE, Murray KM, Hershey DD, et al. Developing a Patient-Centered mHealth App: A Tool for Adolescents With Type 1 Diabetes and Their Parents. *JMIR Mhealth Uhealth*. Apr 19 2017;5(4):e53. doi:10.2196/mhealth.6654

50. Houlihan BV, Coleman C, Kuo DZ, Plant B, Comeau M. What Families of Children With Medical Complexity Say They Need: Humanism in Care Delivery Change. *Pediatrics*. Jan 1 2024;153(Suppl 1)doi:10.1542/peds.2023-063424F

51. Huggett D, Flynn A, Jaouich A, Taylor-Gates M, Davidson S. Engaging Youth in a Mental Health System Improvement Initiative in Ontario: Developing the Be Safe Resource. *Canadian Journal of Community Mental Health*. 2017;36(2):121–131. doi:10.7870/cjcmh-2017-019

52. Keir M, Tarr C, McFadden C, et al. Determining Research Priorities With Teen and Adult Congenital Heart Disease Patients: A Mixed-Methods Study. *CJC Pediatr Congenit Heart Dis*. Apr 2023;2(2):74–81. doi:10.1016/j.cjcpc.2022.12.002

53. Kim L, Costello C, Golding MA, Janse van Rensburg C, Protudjer JLP, Wittmeier K. Determining the benefits and drawbacks of parents using personal connections and social networks for recruitment in research projects: a qualitative study. *Res Involv Engagem*. Jul 26 2023;9(1):58. doi:10.1186/s40900-023-00470-1

54. Kim L, Hermansen AM, Cook K, Siden H. Exploring What Motivates Parents of Children Living With Medical Complexity to Participate in Research. *Child Care Health Dev*. Sep 2024;50(5):e13331. doi:10.1111/cch.13331

55. Kirk S, Fraser C, Evans N, et al. Perceptions of the key components of effective, acceptable and accessible services for children and young people experiencing common mental health problems: a qualitative study. *BMC Health Serv Res*. Apr 24 2023;23(1):391. doi:10.1186/s12913-023-09300-2

56. Knochel K, Zaimovic V, Gatzweiler B, et al. Participatory Development of a Modular Advance Care Planning Program in Pediatric Palliative Care (MAPPS). *J Pain Symptom Manage*. Feb 2022;63(2):189–198. doi:10.1016/j.jpainsymman.2021.09.013

57. Kramer JM, Schwartz AE. Development of the Pediatric Disability Inventory-Patient Reported Outcome (PEDI-PRO) measurement conceptual framework and item candidates. *Scand J Occup Ther*. Sep 2018;25(5):335–346. doi:10.1080/11038128.2018.1502344

58. Lakhanpaul M, Culley L, Robertson N, et al. A structured collaborative approach to intervention design using a modified intervention mapping approach: a case study using the Management and Interventions for Asthma (MIA) project for South Asian children. *BMC Med Res Methodol*. Nov 2 2020;20(1):271. doi:10.1186/s12874-020-01148-y

59. Leake HB, Heathcote LC, Ferencz N, et al. Codesign and Evaluation of Pain Science Messages on Social Media With Adolescents With a History of Chronic Pain. *J Pain*. Sep 2024;25(9):104580. doi:10.1016/j.jpain.2024.104580

60. Leblond M, Galati M, Roberts J, et al. Co-Creating the Experience of Consent for Newborn Genome Sequencing: The Generation Study. *Public Health Genomics*. 2024;27(1):210–227. doi:10.1159/000541935

61. Liu FF, Lew A, Andes E, et al. Implementation strategies for depression and anxiety screening in a pediatric cystic fibrosis center: A quality improvement project. *Pediatr Pulmonol*. Dec 2020;55(12):3328–3336. doi:10.1002/ppul.24951

62. Livanou M, Bull M, Manitsa I, Hunt J, Lane R, Heneghan A. Co-producing a complex psychosocial intervention during COVID-19 with young people transitioning from adolescent secure hospitals to adult services in England: Moving Forward intervention (MFi). *Child Adolesc Ment Health*. Feb 2024;29(1):43–55. doi:10.1111/camh.12667

63. Lopez-Vargas P, Tong A, Crowe S, et al. Research priorities for childhood chronic conditions: a workshop report. *Arch Dis Child*. Mar 2019;104(3):237–245. doi:10.1136/archdischild-2018-315628

64. Ludlow K, Russell JK, Ryan B, et al. Co-designing a digital mental health platform, "Momentum", with young people aged 7-17: A qualitative study. *Digit Health*. Jan–Dec 2023;9:20552076231216410. doi:10.1177/20552076231216410

65. Macarthur C, Walsh CM, Buchanan F, et al. Development of the patient-oriented research curriculum in child health (PORCCH). *Research Involvement and Engagement*. 2021/05/10 2021;7(1):27. doi:10.1186/s40900-021-00276-z

66. Mackworth-Young CR, Dringus S, Dauya E, et al. Putting youth at the centre: co-design of a community-based intervention to improve HIV outcomes among youth in Zimbabwe. *Wellcome Open Res*. 2022;7:53. doi:10.12688/wellcomeopenres.17531.2

67. Manning JC, Hemingway P, Redsell SA. Survived so what? Identifying priorities for research with children and families post-paediatric intensive care unit. *Nurs Crit Care*. Mar 2018;23(2):68–74. doi:10.1111/nicc.12298

68. Martin EG, Rasiah J, Claassen CS, et al. Engaging youth and parents in clinical pediatric research: A case-based example. *Paediatr Child Health*. Jul 2023;28(4):235–240. doi:10.1093/pch/pxac111

69. Massey J, Tsianakas V, Gordon A, Sadler N, Robert G. Co-designing complex therapy interventions with parents as partners in the care of children with cerebral palsy: An Experience-based Co-design study in England. *Res Dev Disabil*. Aug 2024;151:104793. doi:10.1016/j.ridd.2024.104793

70. McKenna S, Hutcheon A, Gorban C, Song Y, Scott E, Hickie I. Youth Perspectives on ‘Highly Personalised and Measurement-Based Care’: Qualitative Co-Design of Education Materials. *Health Expectations*. 2024;27(4):e14137. doi:https://doi.org/10.1111/hex.14137

71. McPherson AC, Ball GD, Maltais DB, et al. A Call to Action: Setting the Research Agenda for Addressing Obesity and Weight-Related Topics in Children with Physical Disabilities. *Child Obes*. Feb 2016;12(1):59–69. doi:10.1089/chi.2015.0119

72. Meagher CE, Kariyawasam DS, Concepcion KAE, et al. Codesign and evaluation of advanced therapeutic information resources for and with families of children with neurological conditions: a mixed methods cross-sectional study. *Archives of Disease in Childhood*. 2024;110(4):308–315. doi:10.1136/archdischild-2024-327914

73. Micsinszki SK, Tanel NL, Kowal J, et al. Codesigning simulations and analyzing the process to ascertain principles of authentic and meaningful research engagement in childhood disability research. *Res Involv Engagem*. Nov 9 2022;8(1):60. doi:10.1186/s40900-022-00398-y

74. Morris C, Simkiss D, Busk M, et al. Setting research priorities to improve the health of children and young people with neurodisability: a British Academy of Childhood Disability-James Lind Alliance Research Priority Setting Partnership. *BMJ Open*. Jan 28 2015;5(1):e006233. doi:10.1136/bmjopen-2014-006233

75. Narula P, Gasparetto M, Wong C, et al. Top 10 research priorities for digital technology for adolescents and young persons with inflammatory bowel disease: Results of a James Lind Alliance Priority Setting Partnership. *J Pediatr Gastroenterol Nutr*. Mar 2024;78(3):670–676. doi:10.1002/jpn3.12105

76. Nguyen L, Jack SM, Davis H, et al. Being a sibling of a youth with a neurodisability: A qualitative study about the roles and responsibilities during the transition to adulthood. *Child Care Health Dev*. Mar 2024;50(2):e13241. doi:10.1111/cch.13241

77. Ostojic K, Karem I, Dee-Price BJ, et al. Development of a new social prescribing intervention for families of children with cerebral palsy. *Dev Med Child Neurol*. Feb 2025;67(2):223–234. doi:10.1111/dmcn.16039

78. Oulton K, Gibson F, Williams A, Geoghegan S, Aldiss S, Wray J. Supporting families of children with an undiagnosed genetic condition: Using co-design to ensure the right person is in the right post doing the right job. *Child Care Health Dev*. May 2021;47(3):300–310. doi:10.1111/cch.12838

79. Pagel C, Brown KL, McLeod I, et al. Selection by a panel of clinicians and family representatives of important early morbidities associated with paediatric cardiac surgery suitable for routine monitoring using the nominal group technique and a robust voting process. *BMJ Open*. May 29 2017;7(5):e014743. doi:10.1136/bmjopen-2016-014743

80. Pearson H, Bell C, Cox K, et al. Integration of patient and public involvement in a doctoral research study using the research cycle. *Res Involv Engagem*. Aug 9 2024;10(1):87. doi:10.1186/s40900-024-00620-z

81. Pembroke S, Cody D, Roche EF, et al. Encouraging adolescents' participation during paediatric diabetes clinic visits: Design and development of a question prompt list intervention. *Diabetes Res Clin Pract*. Aug 2022;190:109985. doi:10.1016/j.diabres.2022.109985

82. Pembroke S, Roche EF, Sleath B, et al. Developing a video intervention to improve youth question-asking and provider education during paediatric diabetes clinic encounters: The Promoting Adolescents Communication and Engagement study. *Patient Educ Couns*. Sep 2021;104(9):2170–2176. doi:10.1016/j.pec.2021.02.021

83. Phillips B, Depani S, Morgan J. What do families want to improve in the management of paediatric febrile neutropenia during anti-cancer treatment? Report of a patient/public involvement group. *BMJ Paediatr Open*. 2019;3(1):e000398. doi:10.1136/bmjpo-2018-000398

84. Poku BA, Pilnick A. Research knowledge transfer to improve the care and support of adolescents with sickle cell disease in Ghana. *Health Expect*. Oct 2022;25(5):2515–2524. doi:10.1111/hex.13573

85. Potter E, Lewis C, Tilbury A, Tong J, Sipanoun P. Improving healthcare transition for young people with cancer: factors fundamental to the quality improvement journey. *Br J Nurs*. Jul 4 2024;33(13):622–629. doi:10.12968/bjon.2023.0146

86. Radford C, Slater PJ, Fielden P. The Oncology Family Forum: Collaborating With Families to Codesign Pediatric Oncology Services. *J Patient Exp*. Dec 2020;7(6):1671–1677. doi:10.1177/2374373520950977

87. Randolph G, Coleman C, Allshouse C, Plant B, Kuo DZ. Measuring What Matters to Children With Medical Complexity and Their Families. *Pediatrics*. Jan 1 2024;153(Suppl 1)doi:10.1542/peds.2023-063424C

88. Samuel SM, Takano T, Scott S, et al. Setting New Directions for Research in Childhood Nephrotic Syndrome: Results From a National Workshop. *Can J Kidney Health Dis*. 2017;4:2054358117703386. doi:10.1177/2054358117703386

89. Schwartz AE, Kramer JM. Inclusive approaches to developing content valid patient-reported outcome measure response scales for youth with intellectual/developmental disabilities. *Br J Learn Disabil*. Mar 2021;49(1):100–110. doi:10.1111/bld.12346

90. Seid M, Dellal G, Peterson LE, et al. Co-Designing a Collaborative Chronic Care Network (C3N) for Inflammatory Bowel Disease: Development of Methods. *JMIR Hum Factors*. Feb 22 2018;5(1):e8. doi:10.2196/humanfactors.8083

91. Seko Y, Oh A, Thompson L, Bowman LR, Curran CJ. Transitions Pop-ups: Co-designing client-centred support for disabled youth transitioning to adult life. *Front Rehabil Sci*. 2024;5:1286875. doi:10.3389/fresc.2024.1286875

92. Sheikhan NY, Henderson JL, Halsall T, et al. Stigma as a barrier to early intervention among youth seeking mental health services in Ontario, Canada: a qualitative study. *BMC Health Services Research*. 2023/01/26 2023;23(1):86. doi:10.1186/s12913-023-09075-6

93. Sligo J, Jones B, Davies C, et al. The experiences of young people with chronic illness in New Zealand: A qualitative study. *Child Care Health Dev*. Sep 2019;45(5):660–669. doi:10.1111/cch.12696

94. Sood E, Gramszlo C, Perez Ramirez A, et al. Partnering With Stakeholders to Inform the Co-Design of a Psychosocial Intervention for Prenatally Diagnosed Congenital Heart Disease. *J Patient Exp*. 2022;9:23743735221092488. doi:10.1177/23743735221092488

95. Taylor H, Pennington L, Morris C, et al. Developing the FEEDS toolkit of parent-delivered interventions for eating, drinking and swallowing difficulties in young children with neurodisability: findings from a Delphi survey and stakeholder consultation workshops. *BMJ Paediatr Open*. Apr 2022;6(1)doi:10.1136/bmjpo-2022-001425

96. Tsang VWL, Fletcher S, Thompson C, Smith S. A novel way to engage youth in research: evaluation of a participatory health research project by the international children’s advisory network youth council. *International Journal of Adolescence and Youth*. 2020/12/31 2020;25(1):676–686. doi:10.1080/02673843.2020.1716817

97. Turuba R, Amarasekera A, Howard AM, et al. A qualitative study exploring how young people perceive and experience substance use services in British Columbia, Canada. *Substance Abuse Treatment, Prevention, and Policy*. 2022/05/28 2022;17(1):43. doi:10.1186/s13011-022-00456-4

98. van Schelven F, Boeije H, Rademakers J. Evaluating meaningful impact of Patient and Public Involvement: A Q methodology study among researchers and young people with a chronic condition. *Health Expect*. Apr 2022;25(2):712–720. doi:10.1111/hex.13418

99. van Schelven F, van Weele M, van der Meulen E, Wessels E, Boeije H. Patient and public involvement in the development of the digital tool MyBoT to support communication between young people with a chronic condition and care providers. *Health Expect*. Apr 2024;27(2):e14003. doi:10.1111/hex.14003

100. Vella-Baldacchino M, Perry DC, Roposch A, et al. Research priorities in children requiring elective surgery for conditions affecting the lower limbs: a James Lind Alliance Priority Setting Partnership. *BMJ Open*. Dec 30 2019;9(12):e033233. doi:10.1136/bmjopen-2019-033233

101. Verwoerd A, Armbrust W, Cowan K, et al. Dutch patients, caregivers and healthcare professionals generate first nationwide research agenda for juvenile idiopathic arthritis. *Pediatr Rheumatol Online J*. Apr 7 2021;19(1):52. doi:10.1186/s12969-021-00540-2

102. Vickers D, Salorio-Corbetto M, Driver S, et al. Involving Children and Teenagers With Bilateral Cochlear Implants in the Design of the BEARS (Both EARS) Virtual Reality Training Suite Improves Personalization. *Front Digit Health*. 2021;3:759723. doi:10.3389/fdgth.2021.759723

103. Virk P, Laskin S, Gokiert R, et al. MyHEARTSMAP: development and evaluation of a psychosocial self-assessment tool, for and by youth. *BMJ Paediatr Open*. 2019;3(1):e000493. doi:10.1136/bmjpo-2019-000493

104. Walker E, Shaw E, Nunns M, Moore D, Thompson Coon J. No evidence synthesis about me without me: Involving young people in the conduct and dissemination of a complex evidence synthesis. *Health Expect*. May 2021;24 Suppl 1(Suppl 1):122–133. doi:10.1111/hex.13078

105. Walsh CM, Jones NL, McCreath GA, et al. Codevelopment and usability testing of Patient Engagement 101: a Patient-Oriented Research Curriculum in Child Health e-learning module for health care professionals, researchers and trainees. *CMAJ Open*. Oct–Dec 2022;10(4):E872–e881. doi:10.9778/cmajo.20210336

106. Wambua GN, Musindo O, Machuka J, Kumar M. Engagement and partnership with peer mentors in the development of the "Positive and Healthy Living Program": a process paper. *AIDS Care*. Sep 2019;31(9):1145–1151. doi:10.1080/09540121.2019.1574333

107. Webbe JWH, Duffy JMN, Afonso E, et al. Core outcomes in neonatology: development of a core outcome set for neonatal research. *Arch Dis Child Fetal Neonatal Ed*. Jul 2020;105(4):425–431. doi:10.1136/archdischild-2019-317501

108. Weiler-Wichtl LJ, Leiss U, Gojo J, et al. Good to know - This is PPIE! Development of a training tool for public and patient involvement and engagement in pediatric oncological research. *Cancer Rep (Hoboken)*. Jun 2023;6(6):e1835. doi:10.1002/cnr2.1835

109. Weiler-Wichtl LJ, Schneider C, Gsell H, et al. Asking those who know their needs best: A framework for active engagement and involvement of childhood cancer survivors and parents in the process of psychosocial research-A workshop report. *Cancer Rep (Hoboken)*. May 2024;7(5):e2071. doi:10.1002/cnr2.2071

110. Wintels SC, Smits DW, van Wesel F, Verheijden J, Ketelaar M. How do adolescents with cerebral palsy participate? Learning from their personal experiences. *Health Expect*. Dec 2018;21(6):1024–1034. doi:10.1111/hex.12796

111. Wong W, Ming D, Pateras S, et al. Outcomes of End-User Testing of a Care Coordination Mobile App With Families of Children With Special Health Care Needs: Simulation Study. *JMIR Form Res*. Aug 28 2023;7:e43993. doi:10.2196/43993

112. Wood MD, West NC, Fokkens C, et al. An Individualized Postoperative Pain Risk Communication Tool for Use in Pediatric Surgery: Co-Design and Usability Evaluation. *JMIR Pediatr Parent*. Nov 17 2023;6:e46785. doi:10.2196/46785

113. Xafis V, Gillam L, Hynson J, Sullivan J, Cossich M, Wilkinson D. Caring Decisions: The Development of a Written Resource for Parents Facing End-of-Life Decisions. *J Palliat Med*. Nov 2015;18(11):945–55. doi:10.1089/jpm.2015.0048
