# Supplementary material for: The Many Faces of Engagement: A Scoping Review of Paediatric Patient and Family Engagement in Clinical Care, Education and Research
Source: Health Expect. 2026 Aug 18;29(4):e70756. doi: 10.1111/hex.70756 (PMC13486972; doi:10.1111/hex.70756)
Supplement: Supplementary file 4 [file HEX-29-e70756-s004.docx]

**Appendix B:** Revisions Made to Exclusion Criteria

| **Initial Exclusion Criteria** | **Revisions/Additions** | **Final Exclusion Criteria** |
| --- | --- | --- |
| Abstract-only or full-text not available |  | Abstract-only or full-text not available |
| Published before 2011 |  | Published before 2011 |
| Non-primary studies (e.g., narrative reviews, scoping reviews, protocol, conference proceedings) | - Include reviews of any type if patients/family members were involved as collaborators in the review - Include commentaries/viewpoints if authors reporting on patient/family engagement within their own research or project | Non-primary studies that do not involve patients/family members as collaborators or do not report on their own engagement initiatives |
| Non-healthcare related topic | - Exclude health promotion or disease prevention efforts (e.g., vaccination, sexually transmitted infection education) | Non-healthcare related topic, including health promotion/disease prevention efforts |
| Not focused on children and youth (aged 4-18 years) | - Include studies focused on those aged 0-24 years - Include studies focused on parents/family members of children/youth aged 0-24 years - Exclude studies where mean age of patients is over 24 years old (with the exception of former patients) | Not focused on children and youth (aged 0-24 years) or their parents/family members |
| Not conducted in a pediatric institution/setting | - Include studies that are relevant to a pediatric institution/setting (e.g., has clinical implications) | Not relevant to a pediatric institution/setting |
| No description of explicit patient/family member engagement throughout the course of the study | - Exclude studies when those being engaged do not have lived experience or a connection to issue being investigated - Include studies where engagement happens at a single point in time (e.g., consultation/inform) or an ongoing basis - Exclude studies that use inherently participatory methods (e.g., photovoice) | No description of explicit patient/family member engagement with lived experience at any point during the study |
