# Supplementary material for: The Many Faces of Engagement: A Scoping Review of Paediatric Patient and Family Engagement in Clinical Care, Education and Research
Source: Health Expect. 2026 Aug 18;29(4):e70756. doi: 10.1111/hex.70756 (PMC13486972; doi:10.1111/hex.70756)
Supplement: Supplementary file 5 [file HEX-29-e70756-s005.docx]

**Appendix A:** Literature Search Strategy

| **Date** | November 22, 2024 |
| --- | --- |
| **Topic** | Pediatric patient and family engagement in healthcare delivery and research |
| **Date limit(s)** | 2011-2024 |
| **Language** | English |

| **Database [Platform]** *revised search run November 22, 2024* | **Results** |
| --- | --- |
| Ovid MEDLINE(R) ALL 1946 to November 21, 2024 | 5952 |
| Ovid Embase Classic+Embase <1947 to 2024 Week 46> | 5209 |
| Ovid APA PsycINFO 1806- to November 2024 Week 3> | 2560 |
| Ebscohost CINAHL Plus – November 22, 2024 | 1556 |
| Clarivate Web of Science Core Collection – November 22, 2024 | 1540 |
| **TOTAL** | **16,817** |
| **Deduplicated total (5842 duplicates removed in Covidence)** | 10975 |

Search strategy:

Ovid MEDLINE(R) ALL 1946 to November 21, 2024

| **#** | **Searches** | **Results** |
| --- | --- | --- |
| 1 | "Delivery of Health Care"/ or Decision making/ or *Health occupations/ed or (((deliver* or system* or provider? or user? or patient?) adj2 (healthcare or "health care")) or "healthcare" or "health care" or decision making or communicat* or health* service? or health* system? or clinical trainee? or "competency-based education" or "educational material? " or "educational intervention?" or "educational resource?" or "medical education").tw,kf. | 1737459 |
| 2 | *Patient Participation/ or (patient-centred research or patient-centered research or "patients as? expert?" or "expert patient?" or ((patient? or youth or consumer? or youth-adult or Y-AP or p?ediatric* or family or families or adolescen* or teen* or child* or young adult?) adj3 (engag* or partner* or involv* or collaborat* or particip*))).tw,kf. | 321219 |
| 3 | Community-Based Participatory Research/mt or *Health Services Research/ or (research design* or research method* or research partner* or research personnel* or investigator? or researcher? or Community-Based Participatory Research* or CBPR or PAR or action research* or participative action research* or Consumer-Driven research* or community action-based research* or Research Technique* or research prioriti?ation or Health Care Research* or healthcare research* or cocreat* or codesign* or co-creat* or co-design* or "reciprocal learning" or "bedside teaching" or "bedside learning" or "illness experience" or "lived experience").tw,kf. | 555306 |
| 4 | *pediatrics/ or Adolescent/ or parents/ or family/ or (adolescen* or teen* or youth* or young person or young people or highschool* or high-school* or patient?).tw,kf. | 10305504 |
| 5 | 1 and 2 and 3 and 4 | 7198 |
| 6 | limit 5 to (address or autobiography or bibliography or biography or clinical trial, veterinary or clinical trial protocol or collected work or comment or congress or dataset or dictionary or directory or duplicate publication or editorial or english abstract or electronic supplementary materials or historical article or interactive tutorial or interview or introductory journal article or lecture or legal case or legislation or letter or news or newspaper article or observational study, veterinary or overall or patient education handout or periodical index or personal narrative or portrait or randomized controlled trial, veterinary or retracted publication or "retraction of publication" or video-audio media or webcast) | 261 |
| 7 | 5 not 6 | 6937 |
| 8 | limit 7 to (english language and yr="2011 -Current") | 5980 |
| 9 | remove duplicates from 8 | 5952 |

Ovid Embase Classic+Embase <1947 to 2024 Week 46>

| **#** | **Searches** | **Results** |
| --- | --- | --- |
| 1 | "health care delivery"/ or Decision making/ or *health care personnel/ or (((deliver* or system* or provider? or user? or patient?) adj2 (healthcare or "health care")) or "healthcare" or "health care" or decision making or communicat* or health* service? or health* system? or clinical trainee? or "competency-based education" or "educational material? " or "educational intervention?" or "educational resource?" or "medical education").tw,kf. | 2478050 |
| 2 | *Patient Participation/ or (patient-centred research or patient-centered research or "patients as? expert?" or "expert patient?" or ((patient? or youth or consumer? or youth-adult or Y-AP or p?ediatric* or family or families or adolescen* or teen* or child* or young adult?) adj3 (engag* or partner* or involv* or collaborat* or particip*))).tw,kf. | 456565 |
| 3 | Participatory Research/ or *Health Services Research/ or (research design* or research method* or research partner* or research personnel* or investigator? or researcher? or Community-Based Participatory Research* or CBPR or PAR or action research* or participative action research* or Consumer-Driven research* or community action-based research* or Research Technique* or research prioriti?ation or Health Care Research* or healthcare research* or cocreat* or codesign* or co-creat* or co-design* or "reciprocal learning" or "bedside teaching" or "bedside learning" or "illness experience" or "lived experience").tw,kf. | 660093 |
| 4 | *pediatrics/ or Adolescent/ or parent/ or family/ or (adolescen* or teen* or youth* or young person or young people or highschool* or high-school* or patient?).tw,kf. | 14837386 |
| 5 | 1 and 2 and 3 and 4 | 9891 |
| 6 | limit 5 to (books or chapter or conference abstract or conference paper or "conference review" or data paper or editorial or erratum or letter or note or short survey or tombstone) | 3598 |
| 7 | 5 not 6 | 6293 |
| 8 | limit 7 to (english language and yr="2011 -Current") | 5295 |
| 9 | remove duplicates from 8 | 5209 |

Ovid APA PsycInfo <1806 to November 2024 Week 3>

| **#** | **Searches** | **Results** |
| --- | --- | --- |
| 1 | "health care delivery"/ or Decision making/ or *health personnel/ or (((deliver* or system* or provider? or user? or patient?) adj2 (healthcare or "health care")) or "healthcare" or "health care" or decision making or communicat* or health* service? or health* system? or clinical trainee? or "competency-based education" or "educational material? " or "educational intervention?" or "educational resource?" or "medical education").tw,hw. | 824858 |
| 2 | *Client Participation/ or (patient-centred research or patient-centered research or "patients as? expert?" or "expert patient?" or ((patient? or youth or consumer? or youth-adult or Y-AP or p?ediatric* or family or families or adolescen* or teen* or child* or young adult?) adj3 (engag* or partner* or involv* or collaborat* or particip*))).tw,hw. | 140754 |
| 3 | Action Research/ or (research design* or research method* or research partner* or research personnel* or investigator? or researcher? or Community-Based Participatory Research* or CBPR or PAR or action research* or participative action research* or Consumer-Driven research* or community action-based research* or Research Technique* or research prioriti?ation or Health Care Research* or healthcare research* or cocreat* or codesign* or co-creat* or co-design* or "reciprocal learning" or "bedside teaching" or "bedside learning" or "illness experience" or "lived experience").tw,hw. | 329882 |
| 4 | pediatrics/ or Adolescent health/ or parents/ or family/ or (adolescen* or teen* or youth* or young person or young people or highschool* or high-school* or patient?).tw,hw,mh. | 1563252 |
| 5 | 1 and 2 and 3 and 4 | 3390 |
| 6 | limit 5 to ("0200 book" or "0240 authored book" or "0280 edited book" or "0300 encyclopedia" or "0400 dissertation abstract" or "0500 electronic collection") | 825 |
| 7 | 5 not 6 | 2565 |
| 8 | remove duplicates from 7 | 2560 |

Ebscohost CINAHL Plus – November 22, 2024

| **#** | **Query** | **Limiters/Expanders** | **Last Run Via** | **Results** |
| --- | --- | --- | --- | --- |
| S1 | MH "health care delivery" or MH Decision making or MM "Health Occupations/ED" or TX (((deliver* or system* or provider? or user? or patient?) W2 (healthcare or "health care")) or "healthcare" or "health care" or decision making or communicat* or health* service? or health* system? or clinical trainee? or "competency-based education" or "educational material? " or "educational intervention?" or "educational resource?" or "medical education") | Search modes - Proximity | Interface - EBSCOhost Research Databases  Search Screen - Advanced Search  Database - CINAHL Plus with Full Text | 2,439,569 |
| S2 | MM Patient Participation or TX (patient-centred research or patient-centered research or "patients as? expert?" or "expert patient?" or ((patient? or youth or consumer? or youth-adult or Y-AP or p?ediatric* or family or families or adolescen* or teen* or child* or young adult?) W3 (engag* or partner* or involv* or collaborat* or particip*))) | Search modes - Proximity | Interface - EBSCOhost Research Databases  Search Screen - Advanced Search  Database - CINAHL Plus with Full Text | 220,941 |
| S3 | MH Action Research or MM Health Services Research OR TX (research design* or research method* or research partner* or research personnel* or investigator? or researcher? or Community-Based Participatory Research* or CBPR or PAR or action research* or participative action research* or Consumer-Driven research* or community action-based research* or Research Technique* or research prioriti?ation or Health Care Research* or healthcare research* or cocreat* or codesign* or co-creat* or co-design* or "reciprocal learning" or "bedside teaching" or "bedside learning" or "illness experience" or "lived experience") | Search modes - Proximity | Interface - EBSCOhost Research Databases  Search Screen - Advanced Search  Database - CINAHL Plus with Full Text | 612,931 |
| S4 | MM pediatrics or MH Adolescent or MH parents or MH family or TX (adolescen* or teen* or youth* or young person or young people or highschool* or high-school* or patient?) | Search modes - Proximity | Interface - EBSCOhost Research Databases  Search Screen - Advanced Search  Database - CINAHL Plus with Full Text | 3,627,284 |
| S5 | S1 AND S2 AND S3 AND S4 | Search modes - Proximity | Interface - EBSCOhost Research Databases  Search Screen - Advanced Search  Database - CINAHL Plus with Full Text | 70,096 |
| S6 | S1 AND S2 AND S3 AND S4 | Limiters - Publication Date: 20110101-20241231; English Language; Peer Reviewed; Exclude Pre-CINAHL; Exclude MEDLINE records; Human  Search modes - Proximity | Interface - EBSCOhost Research Databases  Search Screen - Advanced Search  Database - CINAHL Plus with Full Text | 28,202 |
| S7 | S1 AND S2 AND S3 AND S4 | Limiters - Publication Date: 20110101-20241231; English Language; Peer Reviewed; Exclude Pre-CINAHL; Exclude MEDLINE records; Human  Narrow by SubjectAge: - child: 6-12 years  Narrow by SubjectAge: - adolescent: 13-18 years  Search modes - Proximity | Interface - EBSCOhost Research Databases  Search Screen - Advanced Search  Database - CINAHL Plus with Full Text | 1556 |

Clarivate Web of Science Core Collection – November 22, 2024

| 1 | TS=(((deliver* or system* or provider? or user? or patient?) AND (healthcare or "health care")) or "healthcare" or "health care" or decision making or communicat* or health* service? or health* system? or clinical trainee? or "competency-based education" or "educational material? " or "educational intervention?" or "educational resource?" or "medical education") | 3,780,858 |
| --- | --- | --- |
| 2 | TS=(patient-centred research or patient-centered research or "patients as? expert?" or "expert patient?" or ((patient? or youth or consumer? or youth-adult or Y-AP or p?ediatric* or family or families or adolescen* or teen* or child* or young adult?) AND (engag* or partner* or involv* or collaborat* or particip*))) | 2,296,431 |
| 3 | TS=(research design* or research method* or research partner* or research personnel* or investigator? or researcher? or Community-Based Participatory Research* or CBPR or PAR or action research* or participative action research* or Consumer-Driven research* or community action-based research* or Research Technique* or research prioriti?ation or Health Care Research* or healthcare research* or cocreat* or codesign* or co-creat* or co-design* or "reciprocal learning" or "bedside teaching" or "bedside learning" or "illness experience" or "lived experience") | 4,121,772 |
| 4 | TS=(adolescen* or teen* or youth* or young person or young people or highschool* or high-school* or patient?) | 8,891,705 |
| 5 | #4 AND #3 AND #2 AND #1 | 77,462 |
| 6 | ALL=(Conference abstract? or Conference paper? or conference presentation?) | 118,827 |
| 7 | #5 NOT #6 | 76,926 |
| 8 | #5 NOT #6 and Article (Document Types) and English (Languages) and 2011 or 2012 or 2013 or 2014 or 2015 or 2016 or 2017 or 2018 or 2019 or 2020 or 2021 or 2022 or 2023 or 2024 or 2025 (Publication Years) | 54,869 |
| 9 | #5 NOT #6 and Article (Document Types) and English (Languages) and 2011 or 2012 or 2013 or 2014 or 2015 or 2016 or 2017 or 2018 or 2019 or 2020 or 2021 or 2022 or 2023 or 2024 or 2025 (Publication Years) and Pediatrics (Web of Science Categories) | 1540 |

Grey Literature Search String

The initial search string used was:

*patient centred care engagement and research or education and pediatrics*

As this strategy yielded a limited number of relevant results, it was subsequently refined to include the following combinations:

*patient centred care engagement and pediatrics OR*
 *health care delivery pediatrics OR*
 *patient engagement pediatrics OR*
 *patient research pediatrics OR*
 *patient-oriented care AND engagement AND research AND pediatrics OR*
 *((healthcare delivery) AND (community-based participatory research) AND (patient centred care research))*
