# Supplementary material for: The Many Faces of Engagement: A Scoping Review of Paediatric Patient and Family Engagement in Clinical Care, Education and Research
Source: Health Expect. 2026 Aug 18;29(4):e70756. doi: 10.1111/hex.70756 (PMC13486972; doi:10.1111/hex.70756)
Supplement: Supplementary file 6 [file HEX-29-e70756-s006.docx]

**Appendix C:** Methods of Contribution for Partners

| Advisor Name | Group discussions + brainstorming at advisor meetings | Verbal feedback on paper & tables advisor meetings | Written feedback on paper + study materials using shared documents | Full-text screening of articles in Covidence | Developing figures or tables for paper (outside of advisor meetings) | Reviewing+ editing sections of the paper | Writing/editing sections of paper: Results | Writing/editing sections of paper: Discussion | Writing/editing sections of paper: Implications & Conclusion | Developing infographics/knowledge translation tools to reach patients + families | Additional tasks |
| --- | --- | --- | --- | --- | --- | --- | --- | --- | --- | --- | --- |
| Partner #1 | ✓ | ✓ | ✓ | ✗ | ✗ | ✓ | ✓ | ✗ | ✓ | ✓ | ✓ |
| Partner #2 | ✓ | ✓ | ✓ | ✓ | ✓ | ✓ | ✓ | ✓ | ✓ | ✓ | ✓ |
| Partner #3 | ✓ | ✓ | ✓ | ✓ | ✓ | ✓ | ✓ | ✓ | ✓ | ✓ | ✓ |
